# Host cell traversal by *Plasmodium* parasites drives sterilizing T cell-mediated immunity following immunization

**DOI:** 10.1038/s41564-026-02477-2

**Published:** 2026-09-30

**Authors:** Ana Rodrigues, António M. Mendes, Rute Gonçalves, Helena Nunes-Cabaço, Sofia Marques, Nuno Valente-Leal, Daniel Gomes da Costa, Cristina Ferreira, Marc Veldhoen, Miguel Prudêncio, Maria M. Mota, Ângelo Ferreira Chora

**Affiliations:** 1 Gulbenkian Institute for Molecular Medicine, Lisbon, Portugal; 2 Faculdade de Medicina da Universidade de Lisboa, Lisbon, Portugal; 3 Present Address: SGS Portugal S.A., Lisbon, Portugal

**Keywords:** Parasitic infection, Parasite host response

## Abstract

The development of a vaccine capable of conferring sterile protection against *Plasmodium*, the causative agent of malaria, is a major priority for global eradication efforts. Whole-organism vaccines using liver-infective sporozoites provide high levels of sterile protection against pre-erythrocytic infection. Yet, determinants of sporozoite immunogenicity remain poorly characterized. Here we demonstrate, using rodent models of vaccination, that the ability of *Plasmodium* sporozoites to actively migrate through multiple host cells before infecting hepatocytes is required for sterilizing immunity, regardless of the extent of intrahepatic development of immunizing parasites. We further establish host cell traversal as a dominant contributor to the induction of sterile protection. Impaired cell traversal precludes protective liver-resident memory CD8 T cell responses following vaccination but not the production of antiplasmodial antibodies. Our findings show that intrahepatic parasite development alone does not fully account for the magnitude and durability of protection induced by whole-sporozoite vaccination, and highlight parasite behaviour traits as critical immunogenic events shaping sterilizing cellular immunity against *Plasmodium* liver stages.

## Abstract (web summary)

Whole-organism malaria vaccine efficacy relies on host cell traversal by *Plasmodium* parasites, which promotes protective liver-resident CD8 T cell responses independently of intrahepatic parasite development.

## Main

Despite major progress in malaria control over the past two decades, *Plasmodium* infection-associated disease remains a leading cause of morbidity and mortality due to communicable diseases worldwide. Nearly half of the global population lives at risk of *Plasmodium* infection, with the World Health Organization reporting over 282 million cases and 610,000 deaths in 2024 alone^1^. In malaria-endemic regions, natural exposure to *Plasmodium* is non-sterilizing^2^. This persistent susceptibility to re-infection highlights the immunological challenges faced when developing a malaria vaccine capable of conferring complete and durable protection.

From all malaria vaccination strategies targeting the different stages of parasite development, those directed against the pre-erythrocytic stages of *Plasmodium* infection are especially promising^3^. During this obligatory phase of infection, which precedes the onset of blood-stage disease, sporozoites (SPZ) are inoculated by an infected anopheline mosquito, enter the bloodstream and travel to the liver. There, SPZ traverse multiple non-parenchymal and parenchymal cells by repeatedly breaching cell membranes, migrating through the cytoplasm and exiting without establishing infection, in a process termed cell traversal (CT)^4,5^.

So far, two malaria subunit vaccines based on the *Plasmodium falciparum* pre-erythrocytic circumsporozoite protein (CSP) have been licensed for use in endemic settings^6–8^. While these vaccines reduce disease burden, they do not consistently prevent infection^6–9^, a key requirement for eradication. By contrast, whole-sporozoite (W-SPZ) vaccination strategies employing radiation- or genetically attenuated SPZ, or fully infectious SPZ under chemoprophylaxis, induce sterilizing immunity in both humans^10,11^ and rodent models^12^.

While the current view is that this protection largely depends on the capacity of immunizing parasites to infect hepatocytes and sustain prolonged, or even complete, intrahepatic development^13–15^, sterile protection is also achieved with attenuated parasites whose development arrests early after infection^10,16,17^. Now, we show that CT of host cells by immunizing SPZ during the initial steps of infection has a crucial role in shaping protective immunity. We demonstrate that CT is not only required but also a dominant contributor to sterilizing protection after W-SPZ vaccination in rodents by promoting the establishment of *Plasmodium*-specific liver-resident memory CD8 T cells. Our findings open new avenues for rational vaccine design and highlight the need to consider parasite behaviour traits, and not only antigenic load and diversity, for optimizing protective immunity against *Plasmodium*.

## Results

### CT is required for sterile protection after W-SPZ vaccination

**Fig. 1:**
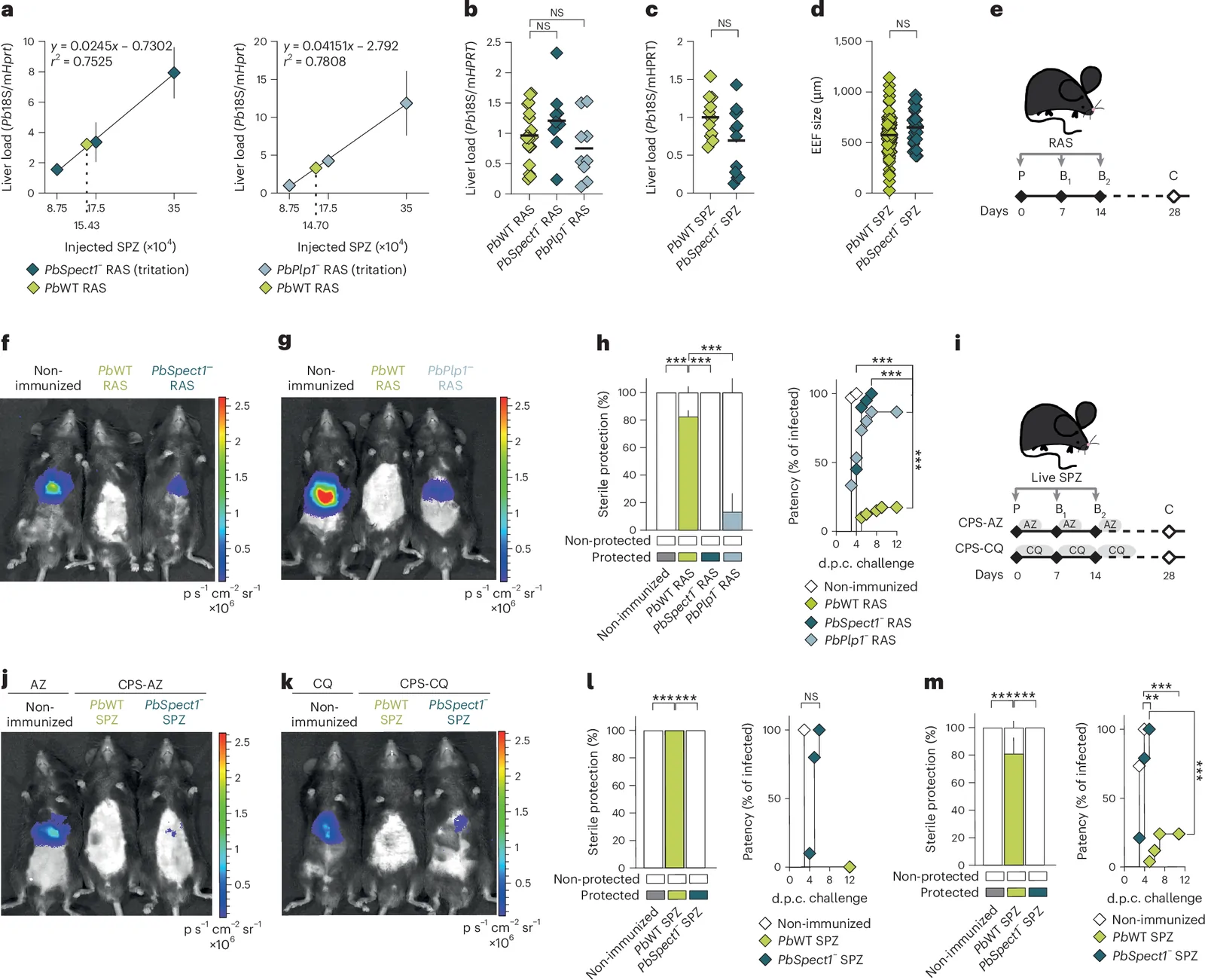
CT motility of immunizing parasites is required for sterilizing immunity against *Plasmodium* hepatic infection. **a**, Linear regression of *Plasmodium* 18S rRNA levels in the livers of C57BL/6 mice, 16 h after receiving the indicated amounts of *PbSpect1*^−^ (*n* = 3 per dose) (left) or *PbPlp1*^−^ (*n* = 3 per dose) (right) or 10^4^ *Pb*WT (*n* = 3) RAS. Dotted lines indicate the estimated number of *PbSpect1*^−^ or *PbPlp1*^−^ RAS required to achieve liver infection loads equivalent to that yielded by the injection of 10^4^ *Pb*WT RAS. **b**, Qantification of *Plasmodium* 18S rRNA levels in the livers of C57BL/6 mice, 16 h after receiving 10^4^ *Pb*WT (*n* = 20; four independent experiments), 15 × 10^4^ *PbSpect1*^−^ (*n* = 10; two independent experiments) or 15 × 10^4^ *PbPlp1*^−^ (*n* = 9; two independent experiments) RAS. **c**,**d**, Quantification of *Plasmodium* 18S rRNA levels (**c**) and EEF size (**d**) in the livers of C57BL/6 mice, 42 h after inoculation of 10^4^ *Pb*WT (*n* = 11) or 15 × 10^4^ *PbSpect1*^−^ (*n* = 12) live SPZ (three independent experiments). **e**, Protocol of RAS immunizations and challenge. B_1_, first boost; B_2_, second boost; C, infectious challenge; P, prime. **f**,**g**, Representative luciferase bioluminescence images of non-immunized and *Pb*WT or *PbSpect1*^−^ (**f**), and non-immunized and *Pb*WT or *PbPlp1*^−^ RAS-immunized C57BL/6 mice (**g**), 48 h after challenge with *Pb*WT SPZ. p, photons; sr, steradian. **h**, Sterile protection, represented as percentage of blood-stage parasite-free mice (left), and time to patency (right) of non-immunized (*n* = 35; seven independent experiments), *Pb*WT (*n* = 40; eight independent experiments), *PbSpect1*^−^ (*n* = 20; 4 independent experiments) or *PbPlp1*^−^ (*n* = 15; three independent experiments) RAS-immunized C57BL/6 mice in the 12-day period after challenge with *Pb*WT SPZ. d.p.c., days post challenge. **i**, Protocol of immunizations with live SPZ under CPS-AZ or CPS-CQ challenge. **j**,**k**, Representative luciferase bioluminescence image of non-immunized and *Pb*WT or *PbSpect1*^−^ CPS-AZ-immunized (**j**) or CPS-CQ-immunized (**k**) C57BL/6 mice, 48 h after challenge with *Pb*WT SPZ. **l**,**m**, Sterile protection, represented as percentage of blood-stage parasite-free mice (left), and time to patency (right) of non-immunized, and *Pb*WT or *PbSpect1*^−^ CPS-immunized C57BL/6 mice under azithromycin (AZ) (**l**) (*n* = 5; one independent experiment, *n* = 15; three independent experiments, and *n* = 10; two independent experiments, respectively) or chloroquine (CQ) (**m**) (*n* = 15; three independent experiments, *n* = 25; four independent experiments and *n* = 19; four independent experiments, respectively) coverage in the 12-day period after challenge with *Pb*WT SPZ. In **a**, data are mean ± s.e.m., with a simple linear regression connecting all data points; in **b**–**d**, data are represented as dot plots, with the black line representing the mean; in **h**,**l** and **m** (left), data are mean  ± s.e.m., and in **h**,**l** and **m** (right), data are the mean day to patency. NS, not significant. *P < 0.05, **P < 0.01, ***P < 0.001, two-sided Mann–Whitney test. For complete statistical analysis, see Source Data Fig 1. Source data

**Extended Data Fig. 1:**
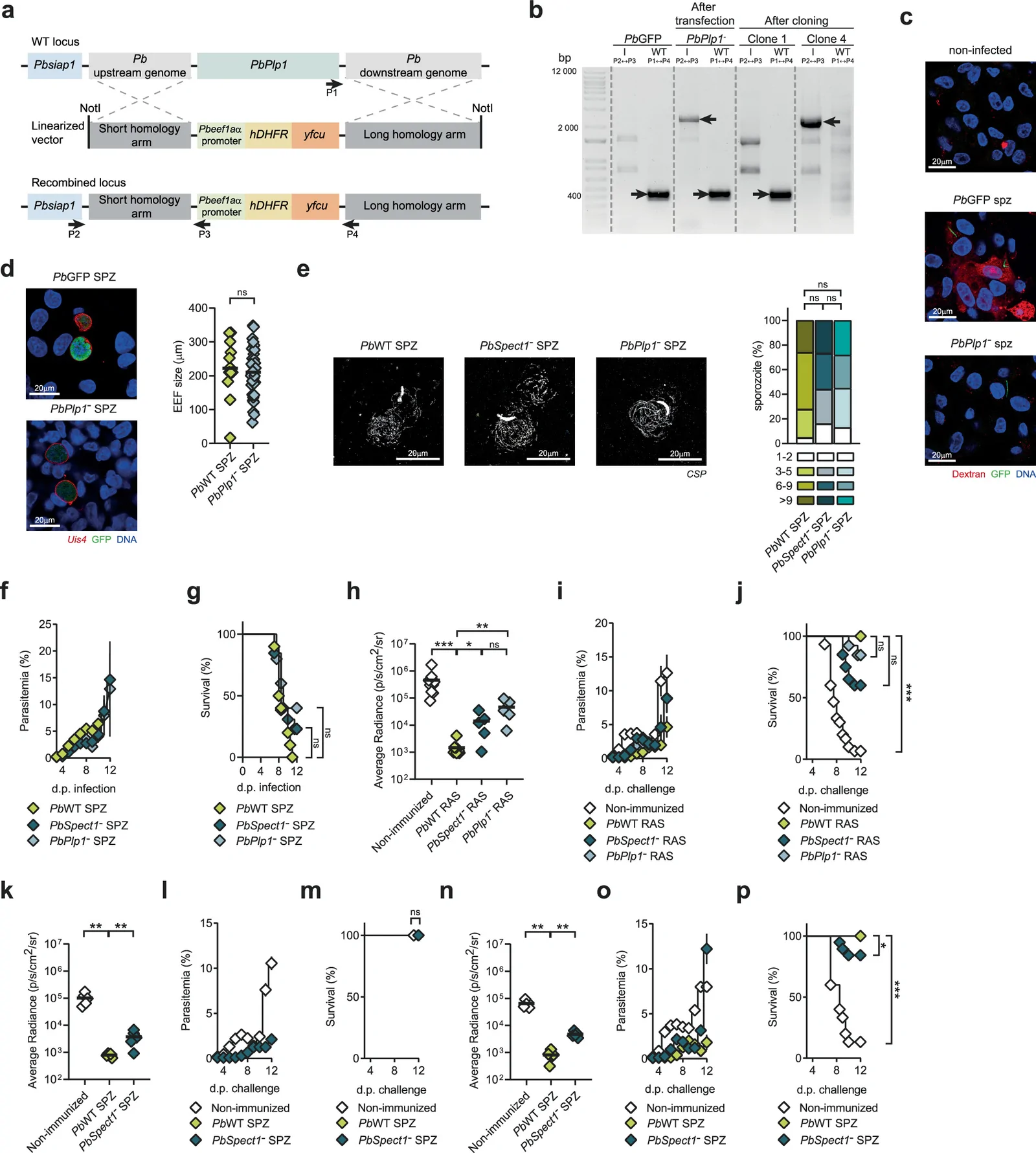
Generation and characterization of the *PbPlp1*- parasite line and clinical outcome following W-SPZ vaccination and challenge. **a**, Schematic of the strategy followed to generate the *PbPlp1*^−^ parasite line, representing the PBANKA_100630 WT locus (top), the PbGEM 293440 linearized vector (middle), and the recombined locus (bottom). Dashed lines represenst homologous recombination sites for the integration of the *Pb*GEM-293340 vector into the *Pb*WT genome. The recombined locus contains a *Pb* elongation factor 1-alpha (*PbEef1aα*) promoter, followed by a resistance cassette composed of the human dihydrofolate reductase (*hDHFR*) gene and the bifunctional yeast enzyme cytosine deaminase and uridyl phosphoribosyl transferase (*yfcu*). Arrows represent the annealing site of primers used for parasite genotyping. **b**, Electrophoretic analysis of *PbPlp1*^−^ parasites after cloning. Bands corresponding to the presence of the *Plp1* wild-type locus are labeled with black arrows pointing right, and bands corresponding to the correct integration of the resistance cassette are labeled with black arrows pointing left (2 independent experiments). **c**, Representative fluorescence confocal microscopy images of HepG2 cells either non-infected (left) or infected, 2 hours earlier with *Pb*GFP (middle) or *PbPlp1*^−^ (right) SPZ in the presence of rhodamine dextran. GFP labels SPZ; rhodamin dextran indicates traversed cells, and Hoescht 33342 stains DNA (2 independent experiments). Images were acquired with 63x magnification and scale bar represents 20 µm. **d**, Representative fluorescence confocal microscopy images (left) and quantification of EEF size (right) of *Pb*GFP (n = 12) or *PbPlp1*^−^ (n = 33) parasites, 48 hours post infection of HepG2 cells. UIS4 labels the parasitophorous vacuole membrane; GFP labels parasite cytoplasm, and Hoescht 33342 stains DNA (3 independent experiments). Images were acquired with 63x magnification and scale bar represents 20 µm. **e**, Representative fluorescence confocal microscopy images (left) and frequency distribution (right) of *Pb*WT (n = 65), *PbSpect1*^−^ (n = 85) or *PbPlp1*^−^ (n = 78) SPZ forming 1-2, 3^−^5, 6-9, or >9 CSP-positive trails during *in vitro* gliding motility assays over a 30-minute period (2 independent experiments). Images were acquired with 40x magnification and scale bar represents 20 µm. **f**, **g** Parasitaemia (**f**) and survival (**g**) in C57BL/6 J after receiving 10^4^ *Pb*WT (n = 10; 2 independent experiments), 15×10^4^ *PbSpect1*^−^ (n = 13; 3 independent experiments) or 15×10^4^ *PbPlp1*^−^ (n = 5; 1 independent experiment) live SPZ. Parasitaemia gating strategy shown in Extended Data Fig. 7a. **h**, Quantification of functional luciferase activity in non-immunized (n = 5), and *Pb*WT (n = 5), *PbSpect1*^−^ (n = 5) or *PbPlp1*^−^ (n = 5) RAS-immunized C57BL/6 J mice, 48 hours after challenge with *Pb*WT SPZ. **i, j** Parasitaemia (**i**) and survival (**j**) in non-immunized (n = 35; 5 independent experiments), and patent *Pb*WT (n = 40; 8 independent experiments), *PbSpect1*^−^ (n = 20; 4 independent experiments) or *PbPlp1*^−^ (n = 15; 3 independent experiments) RAS-immunized C57BL/6 J mice in the 12-days period following challenge with *Pb*WT SPZ. Parasitaemia gating strategy shown in Extended Data Fig. 7a. **k**, Quantification of functional luciferase activity in non-immunized (n = 5), and *Pb*WT (n = 5) or *PbSpect1*^−^ (n = 5) CPS-AZ-immunized C57BL/6 mice, 48 hours after challenge with *Pb*WT SPZ. **l, m** Parasitaemia (**l**) and survival (**m**) in non-immunized (n = 5; 1 independent experiment), and patent *Pb*WT (n = 15; 3 independent experiments) or *PbSpect1*^−^ (n = 10; 2 independent experiments) CPS-AZ-immunized C57BL/6 mice in the 12-days period following challenge with *Pb*WT SPZ. Parasitaemia gating strategy shown in Extended Data Fig. 7a. **n**, Quantification of functional luciferase activity in non-immunized (n = 5), and *Pb*WT (n = 5) or *PbSpect1*^−^ (n = 5) CPS-CQ-immunized C57BL/6 mice, 48 hours after challenge with *Pb*WT SPZ. **o**, **p** Parasitaemia (**o**) and survival (**p**) in non-immunized (n = 15; 3 independent experiments), and patent *Pb*WT (n = 25; 4 independent experiments) or *PbSpect1*^−^ (n = 19; 4 independent experiments) CPS-CQ-immunized C57BL/6 mice in the 12-days period following challenge with *Pb*WT SPZ. Parasitaemia gating strategy shown in Extended Data Fig. 7a. **d**, **h**, **k** and **n**; Data (two-sided Mann-Whitney test) are represented as dot plots, black line represents mean. **e**, Data (Chi-square test) are represented as bars. **f**, **i, l** and **o**; Data (two-sided Mann-Whitney test) are represented as mean percentage of infected erythrocytes ± SEM. **g**, **j**, **m** and **p**; Data (Log rank (Mantel-Cox) test) are represented as mean survival rate. ns – non-significant; * - p < 0.05; ** - p < 0.01; *** - p < 0.001. For complete statistical analysis, please refer to Source Data Extended Data Fig.2. Source data

We sought to compare the immunogenicity of CT-competent *Plasmodium berghei* wild-type (*Pb*WT) parasites with that of CT-deficient parasites lacking the *sporozoite microneme protein essential for cell traversal 1* (*PbSpect1*^−^) gene^18^. To control for potential immunogenic effects of the SPECT1 protein itself, we generated a distinct CT motility-deficient parasite line lacking the *perforin-like protein 1* (*PbPlp1*^−^) gene (Extended Data Fig. 1a,b). As previously reported^19^, *PbPlp1*^−^ parasites failed to traverse HepG2 cells in in vitro cell wounding assays (Extended Data Fig. 1c), while displaying comparable intrahepatic development to their wild-type counterparts (Extended Data Fig. 1d). Assessment of SPZ gliding motility revealed no detectable differences between *Pb*WT, *PbSpect1*⁻ and *PbPlp1*⁻ parasites (Extended Data Fig. 1e), consistent with prior reports showing that defects in CT do not impair SPZ locomotion^18,19^. Both CT-deficient parasite lines exhibit reduced infectivity in vivo^18,19^, complicating the direct comparison of their protective efficacy relative to *Pb*WT parasites at equivalent liver infection loads. To overcome this, the liver loads of increasing inocula of *PbSpect1*^−^ and *PbPlp1*^−^ radiation-attenuated SPZ (RAS) were compared with that resulting from the inoculation of 10^4^ *Pb*WT RAS. This revealed that equivalent parasite loads at 16 h post infection (h.p.i.) required a 15:1 inoculation ratio of *PbSpect1*^−^ or *PbPlp1*^−^ relative to *Pb*WT parasites (Fig. 1a,b). Given that whole-liver quantitative PCR (qPCR) signal at this early stage of infection could, in principle, include contributions from both residual SPZ and early exoerythrocytic forms (EEFs), we validated that the 15:1 ratio of *PbSpect1*⁻ to *Pb*WT fully infectious SPZ resulted in comparable hepatic parasite loads at 42 h.p.i. (Fig. 1c), a stage at which the qPCR signal reflects actively replicating intrahepatic EEFs. Under this condition, both parasite lines displayed comparable intrahepatic development (Fig. 1d). When administered at these adjusted doses, infectious CT-competent and CT-deficient parasites seeded comparable blood-stage infections leading to the establishment of experimental cerebral malaria (ECM) (Extended Data Fig. 1f,g), showing that the different inocula are functionally equivalent and consistent with CT-deficient parasites entering hepatocytes and undergoing productive intrahepatic development in vivo at levels comparable to their wild-type counterparts.

The standardized inocula were subsequently used in a well-established prime–boost–boost RAS immunization protocol in C57BL/6 mice^20^ (Fig. 1e) to evaluate the protective efficacy of each *Pb* line in the 12-day-period after challenge with fully infectious, luciferase-expressing *Pb*WT parasites. In vivo bioluminescence imaging at 48 h post challenge (h.p.c.) revealed that non-immunized mice harboured metabolically active parasites in the liver, whereas *Pb*WT RAS-immunized mice had no detectable bioluminescence activity (Fig. 1f,g and Extended Data Fig. 1h). By constrast, both *PbSpect1*^−^ and *PbPlp1*^−^ RAS-immunized mice displayed higher bioluminescence activity than *Pb*WT RAS-immunized mice, albeit significantly reduced compared with non-immunized mice (Fig. 1f,g and Extended Data Fig. 1h). Sterile protection, assessed by the absence of blood-stage parasites after challenge by bioluminescence, was observed in 82.5% ± 4.5% (mean ± s.e.m.) of the *Pb*WT RAS-immunized cohort, whereas none of *PbSpect1*^−^ and only 13.3% ± 13.3% of *PbPlp1*^−^ RAS-immunized mice remained protected after challenge (Fig. 1h). Erythrocytic infection became patent in unprotected *Pb*WT RAS-immunized mice on day 6.1 ± 0.6 after challenge and on days 4.7 ± 0.2 and 4.2 ± 1.3 in *PbSpect1*^−^ and *PbPlp1*^−^ RAS-immunized mice, respectively (Fig. 1h). The decreased number of parasites seeding the blood stage of infection led to the delayed progression of parasitemia in unprotected mice from all immunized cohorts (Extended Data Fig. 1i), with varying levels of protection from ECM (Extended Data Fig. 1j). Together, these data show that the establishment of sterilizing protection after W-SPZ vaccination requires CT activity by immunizing SPZ, rather than a specific protein mediating the process.

Several studies suggest that the timing of intrahepatic parasite arrest has a major role in the potency of the immune response elicited by vaccination, with longer development of immunizing parasites potentially exposing the host to a wider breadth and quantity of antigens^21,22^. To assess whether CT remained relevant for sterile protection after immunization regimens enabling extended or even complete intrahepatic development of immunizing SPZ, we used two immunization regimens with infectious SPZ under azithromycin or chloroquine chemoprophylaxis (that is, chemoprophylaxis with azithromycin (CPS-AZ)^23^ and chemoprophylaxis with chloroquine (CPS-CQ)^24^) (Fig. 1i). *Pb*WT-immunized mice under both CPS immunization regimens had no detectable metabolically active parasites in the liver at 48 h.p.c. (Fig. 1j,k and Extended Data Fig. 1k,n), with 100% and 80% ± 11.5% of immunized mice remaining blood-stage parasite-free for the 12-day-period after challenge with *Pb*WT SPZ (Fig. 1l,m). By contrast, *PbSpect1*^−^ CPS-AZ- and CPS-CQ-immunized cohorts exhibited only partial reductions in liver stage parasite load (Fig. 1j,k and Extended Data Fig. 1k,n) and failed to achieve sterile protection, with blood-stage infection becoming patent on days 5.1 ± 0.2 and 4.0 ± 0.2 after challenge, respectively (Fig. 1l,m). Unprotected mice, irrespective of receiving CT-competent or CT-deficient immunizing parasites, exhibited delayed parasitemia (Extended Data Fig. 1l,o) and were protected from ECM (Extended Data Fig. 1m,p). Notably, azithromycin also prevented ECM in non-immunized controls (Extended Data Fig. 1m), suggesting that it can counter severe neurological manifestations of malaria. Collectively, these data demonstrate that host CT by immunizing SPZ is a critical determinant of pre-erythrocytic sterilizing immunity after W-SPZ vaccination, regardless of the duration of intrahepatic parasite development.

### CT by immunizing SPZ is a dominant contributor for sterilizing immunity

**Fig. 2:**
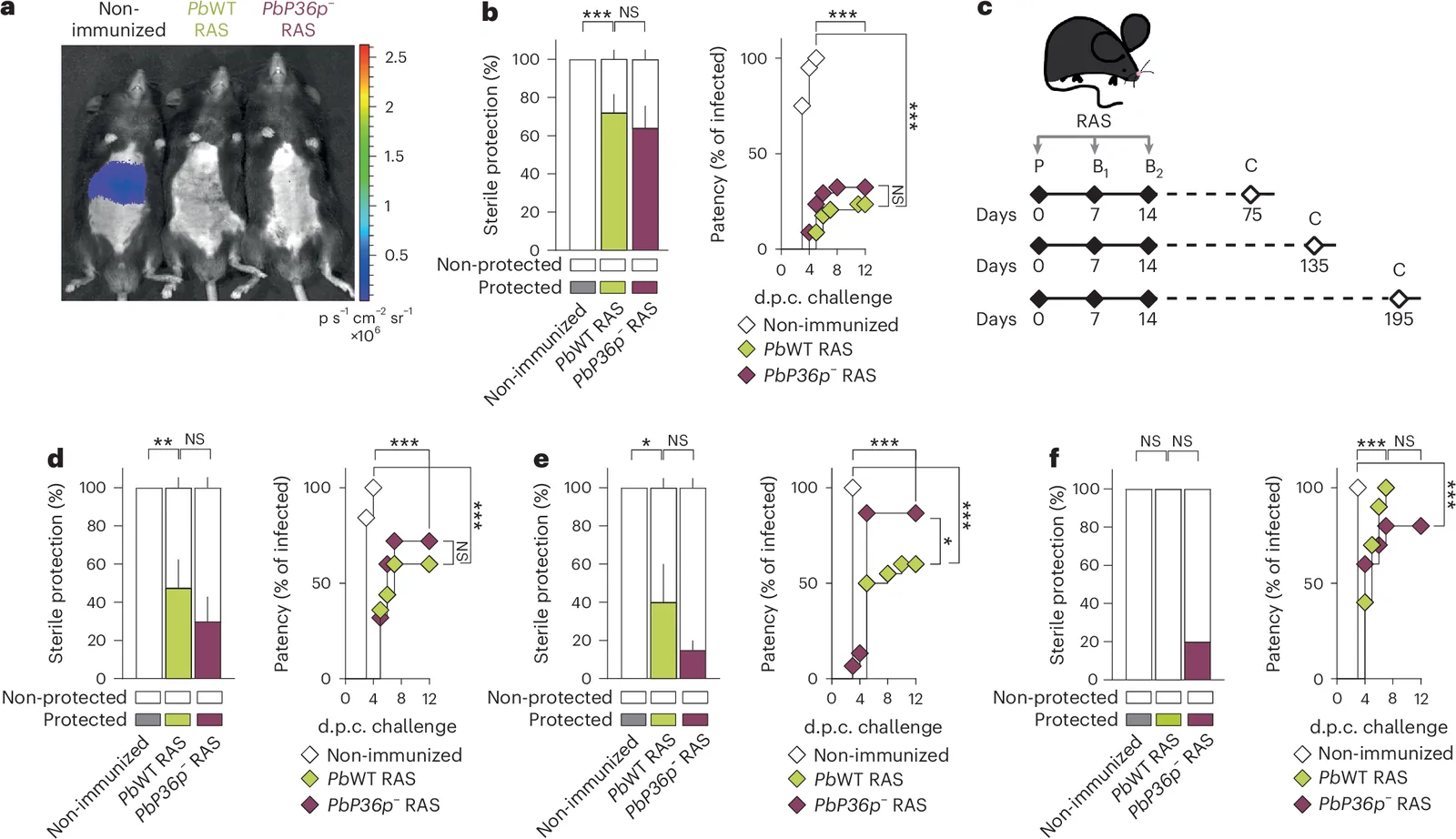
CT motility of immunizing parasites is a dominant contributor for the establishment of sterile protection following W-SPZ RAS vaccination. **a**, Representative luciferase bioluminescence image of non-immunized, and *Pb*WT or *PbP36p*^−^ RAS-immunized C57BL/6J mice, 48 h after challenge with *Pb*WT SPZ. **b**, Sterile protection, represented as a percentage of blood-stage parasite-free mice (left), and time to patency (right) of non-immunized (*n* = 20; four independent experiments), and *Pb*WT (*n* = 34; five idepedent experiments) or *PbP36p*^−^ (*n* = 34; five independent experiments) RAS-immunized C57BL/6 mice in the 12-day period after challenge with *Pb*WT SPZ. **c**, Protocol of RAS immunizations and challenge used to evaluate long-term protection. **d**–**f**, Sterile protection, represented as percentage of blood-stage parasite-free mice (left), and time to patency (right) of non-immunized, and *Pb*WT or *PbP36p*^−^ RAS-immunized C57BL/6 mice in the 12-day period after challenge with *Pb*WT SPZ 75 days (**d**) (*n* = 19, *n* = 25 and *n* = 25, respectively; four independent experiments), 135 days (**e**) (*n* = 12, *n* = 20 and *n* = 15, respectively; two independent experiments) and 195 days (**f**) (*n* = 5, *n* = 10 and *n* = 10, respectively; one independent experiment) after prime. In **b**–**f** (left), data are mean ± s.e.m.; in **b–f** (right), data are the mean day to patency. *P < 0.05, **P < 0.01, ***P < 0.001, two-sided Mann–Whitney test. Source data

**Extended Data Fig. 2:**
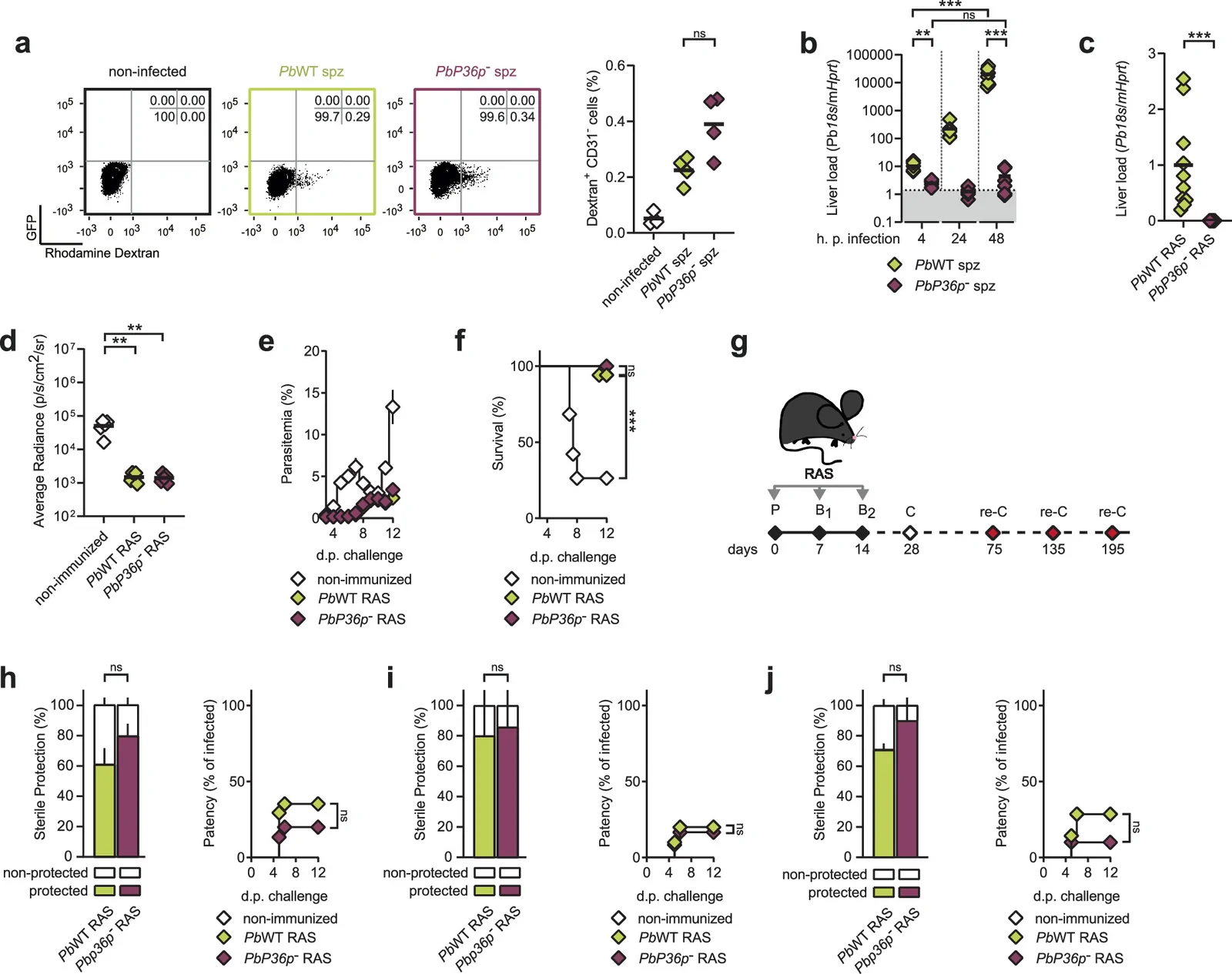
*In vivo* characterization of *PbP36p*- parasite line and sequential challenge protocol. **a**, Representative flow cytometry plots and frequency of traversed, rhodamine dextran-positive liver parenchymal cells in the livers of C57BL/6 mice, either non-infected (n = 3), or 2 hours after receiving 5×10^5^ *Pb*WT (n = 4) or 5×10^5^ *PbP36p*^−^ (n = 4) SPZ (3 independent experiments). Cell gating strategy shown in Extended Data Fig. 7b. **b, c**, Time-dependent quantification of *Plasmodium 18* *s* rRNA levels in the livers of C57BL/6 mice receiving (**b**) 10^4^ *Pb*WT or *PbP36p*^−^ SPZ at 4 (n = 6 per group), 24 (n = 6 and n = 8, respectively) and 48 (n = 6 and n = 8, respectively) hours after infection (2 independent experiments) or (**c**) 10^4^ *Pb*WT (n = 10) or *PbP36p*^−^ (n = 10) RAS at 16 hours after infection (2 independent experiments). Gray area represents unspecific *18* *s* rRNA amplification signal in non-infected controls (n = 5). **d**, Quantification of functional luciferase activity in non-immunized (n = 5), and *Pb*WT (n = 5) or *PbP36p*^−^ (n = 5) RAS-immunized C57BL/6 mice, 48 hours after challenge with *Pb*WT SPZ. **e**, **f** Parasitemia (**e**) and survival (**f**) in non-immunized (n = 20; 4 independent experiments), and patent *Pb*WT (n = 34; 5 independent experiments) or *PbP36p*^−^ (n = 34; 5 independent experiments) RAS-immunized C57BL/6 mice in the 12-days period following challenge with *Pb*WT SPZ. Parasitemia gating strategy shown in Extended Data Fig. 7a. **g**, Protocol used for RAS immunizations to evaluate long-term protection following successive re-challenges. **h**-**j** Sterile protection, represented as percentage of blood stage parasite-free mice (left), and time to patency (right) of *Pb*WT or *PbP36p*^−^ RAS-immunized C57BL/6 mice following re-challenge with *Pb*WT SPZ (**h**) 75 (n = 15 and n = 15, respectively) (**i**) 135 (n = 9 and n = 12, respectively) and (**j**) 195 (n = 7 and n = 10, respectively) days after prime (2 independent experiments). **a-d**, Data (two-sided Mann-Whitney test) are represented as dot plots, black line represents mean. e, Data (two-sided Mann-Whitney test) are represented as mean percentage of infected erythrocytes ± SEM. f, Data (Log rank (Mantel-Cox) test) are represented as mean survival rate. h-j, left panels, Data (two-sided Mann-Whitney test) are represented as bars ± SEM. h-j, right panels, Data (two-sided Mann-Whitney test) are represented as the mean day to patency. ns – non-significant; * - p < 0.05; ** - p < 0.01; *** - p < 0.001. For complete statistical analysis, please refer to Source Data Extended Data Fig.2. Source data

Next, we interrogated the relative contributions of CT and hepatocyte infection for protection by employing *Pb* parasites lacking the *P36p* (*PbP36p*^−^) gene^25^, which retained in vivo CT activity (Extended Data Fig. 2a) but exhibited markedly reduced intrahepatic development, either alive or after irradiation (Extended Data Fig. 2b,c). Both *Pb*WT and *PbP36p*^−^ RAS-immunized mice (Fig. 1e) showed no liver bioluminescence at 48 h.p.c., indicating the successful elimination of hepatic parasites (Fig. 2a and Extended Data Fig. 2d). Consistently, both immunized cohorts exhibited comparable levels of sterile protection (Fig. 2b). Delayed blood-stage onset in unprotected *Pb*WT and *PbP36p*^−^ RAS-immunized mice (Fig. 2b and Extended Data Fig. 2e) correlated with reduced ECM incidence (Extended Data Fig. 2f). Given their comparable levels of short-term sterile protection, we next assessed the durability of protection by challenging *Pb*WT and *PbP36p*^−^ RAS-immunized mice at increasing intervals after priming (Fig. 2c). Both groups displayed a comparable decline in protection over time, with most mice exhibiting patent blood-stage infections when challenged at 195 days after priming (Fig. 2d–f), suggesting that long-term protection wanes at a similar rate regardless of parasite genotype. Nevertheless, *Pb*WT RAS-immunized mice consistently showed slightly higher levels of sterile protection than *PbP36p*⁻ RAS-immunized mice, although this difference did not reach statistical significance. This trend probably reflects additional parasite–host interactions during infection that may subtly influence the magnitude of the response. By contrast, *Pb*WT and *PbP36p*^−^ RAS-immunized mice that remained blood-stage parasite-negative following the initial challenge 28 days after prime (Fig. 2a,b) maintained robust levels of sterile protection upon subsequent consecutive re-challenges at the same timepoints (Extended Data Fig. 2g–j). Together, these observations show that immunization with RAS exhibiting highly reduced intrahepatic development but preserved CT motility can induce sterile protection with comparable durability to their wild-type counterparts, reinforcing that CT is not only required (Fig. 1), but also a major determinant for effective immunity induced by W-SPZ vaccination.

### Lack of CT by SPZ during immunization hinders cellular immunity

**Fig. 3:**
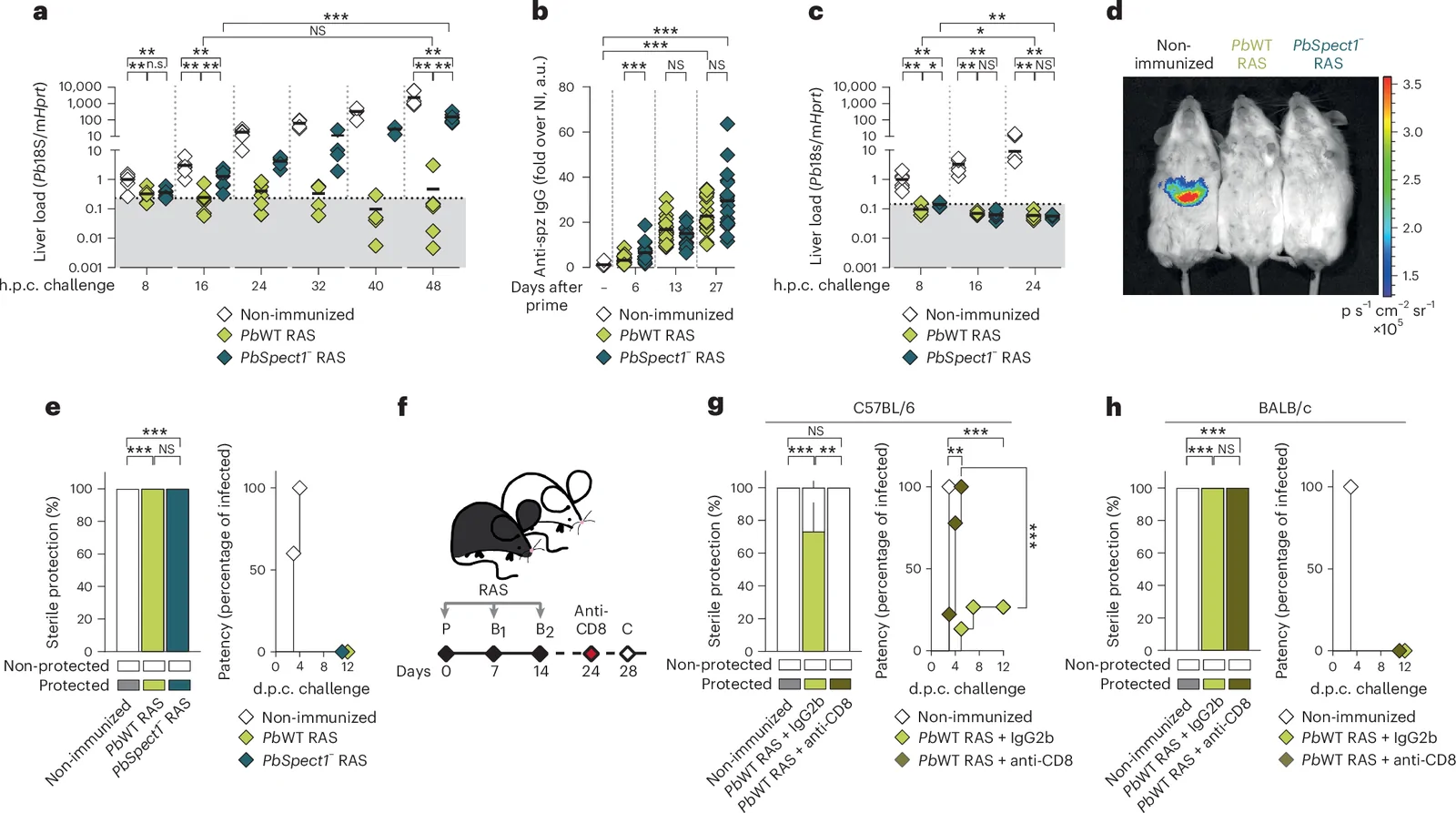
Cellular immunity, but not humoral immunity, is hindered by the lack of host CT during immunization. **a**, Time-dependent quantification of *Plasmodium* 18S rRNA levels in the livers of non-immunized, and *Pb*WT or *PbSpect1*^−^ RAS-immunized C57BL/6J mice at 8 (*n* = 6; two independent experiments), 16 (*n* = 7; two independent experiments), 24 (*n* = 7; two independent experiments), 32 (*n* = 4; one independent experiment), 40 (*n* = 4; one independent experiment) and 48 h (*n* = 8; two independent experiments) after challenge with *Pb*WT SPZ. Grey area represents an 18S rRNA unspecific amplification signal in non-infected controls (*n* = 6; one independent experiment). **b**, Quantification of circulating anti-SPZ IgG levels in non-immunized (NI) (*n* = 12), and *Pb*WT (*n* = 18) or *PbSpect1*^−^ (*n* = 18) RAS-immunized C57BL/6J mice at the indicated days after priming (two independent experiments). **c**, Time-dependent quantification of *Plasmodium* 18S rRNA levels in the livers of non-immunized, and *Pb*WT or *PbSpect1*^−^ RAS-immunized BALB/c mice at 8 (*n* = 6), 16 (*n* = 6) and 24 h (*n* = 6) after challenge with *Pb*WT SPZ. Grey area represents an unspecific 18S rRNA amplification signal in non-infected controls (*n* = 7) (two independent experiments). **d**, Representative luciferase bioluminescence image of non-immunized, and *Pb*WT or *PbSpect1*^−^ RAS-immunized BALB/c mice, 48 h after challenge with *Pb*WT SPZ. **e**, Sterile protection, represented as percentage of blood-stage parasite-free mice (left), and time to patency (right) of non-immunized (*n* = 10), and *Pb*WT (*n* = 10) or *PbSpect1*^−^ (*n* = 10) RAS-immunized BALB/c mice in the 12-day period after challenge with *Pb*WT SPZ (two independent experiments). **f**, Protocol of RAS immunizations and challenge after the administration of CD8 T cell-depleting antibodies. **g**,**h**, Sterile protection, represented as percentage of blood-stage parasite-free mice (left), and time to patency (right) of non-immunized, and *Pb*WT RAS-immunized C57BL/6J (**g**) (*n* = 10; two independent experiments, *n* = 15; three independent experiments and *n* = 8; two independent experiments, respectively) or BALB/c (**h**) (*n* = 5; one independent experiment, *n* = 10; two independent experiments and *n* = 8; two independent experiments, respectively) mice receiving IgG2b or anti-CD8 T cell-depleting antibodies followed by challenge with *Pb*WT SPZ. In **a**–**c**, data are represented as dot plots, with the black line representing the mean. In **e**,**g** and **h** (left), data are mean ± s.e.m.; in **e**,**g** and **h** (right), data are the mean day to patency. *P < 0.05, **P < 0.01, ***P < 0.001, two-sided Mann–Whitney test. Source data

**Extended Data Fig. 3:**
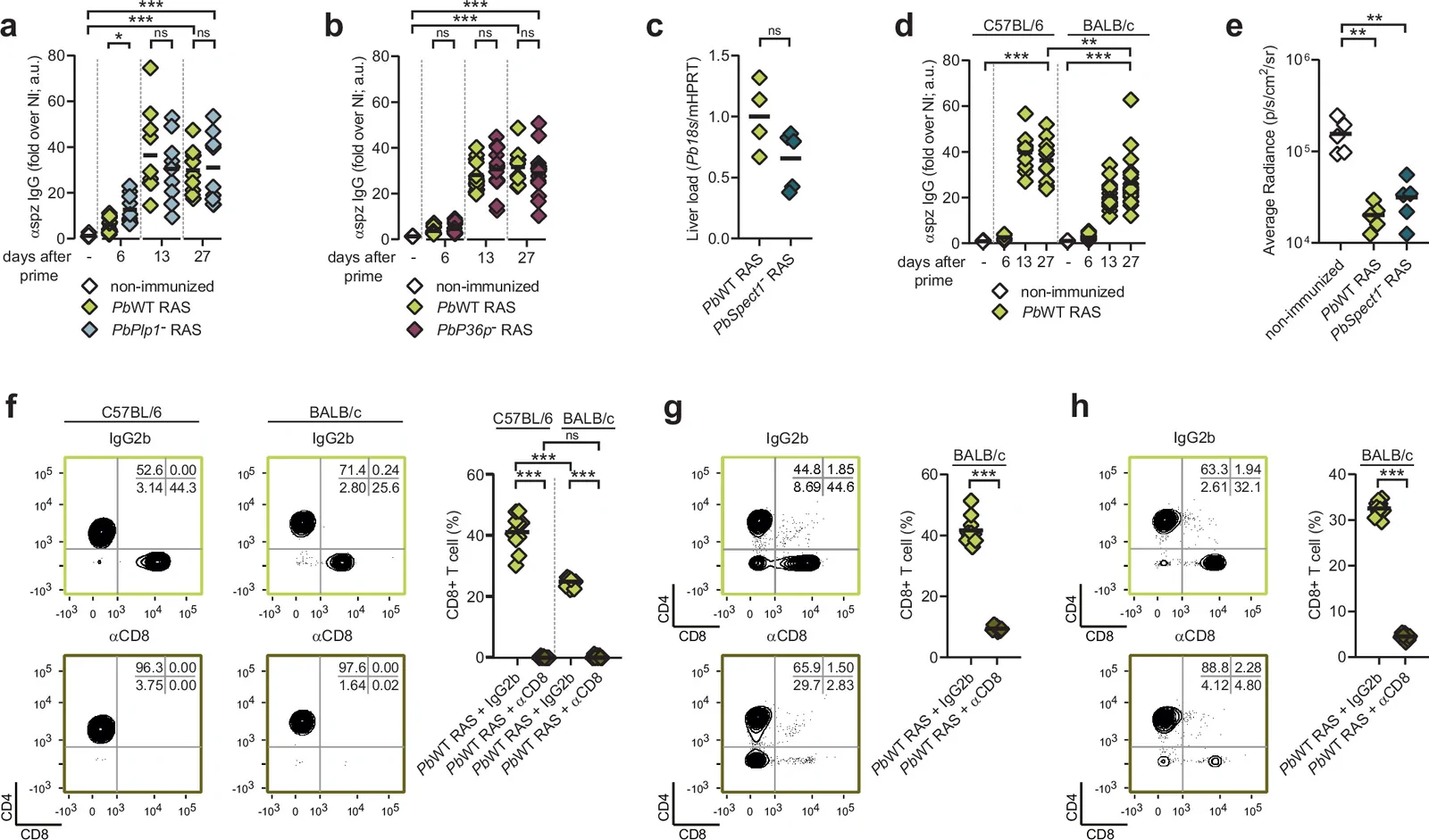
BALB/c humoral responses following W-SPZ vaccination and assessment of CD8 T cell depletion. **a**, Quantification of circulating αSPZ IgG levels in non-immunized (n = 10), and *Pb*WT (n = 10) or *PbPlp1*^−^ (n = 10) RAS-immunized C57BL/6 mice at the indicated days after priming (2 independent experiments). **b**, Quantification of circulating αSPZ IgG levels in non-immunized (n = 5; 1 independent experiment), and *Pb*WT (day 6, n = 14; day 13, n = 15; and day 27, n = 10; 2 independent experiments) or *PbP36p*^−^ (n = 15 per time point; 2 independent experiments) RAS-immunized C57BL/6 J mice at the indicated days after prime. **c**, Quantification of *Plasmodium 18* *s* rRNA levels in the livers of BALB/c mice, 16 hours after inoculation of 10^4^ *Pb*WT (n = 4) or 15×10^4^ *PbSpect1*^−^ (n = 5) RAS (1 experiment). **d**, Quantification of circulating αSPZ IgG levels in C57BL/6 mice, either non-immunized (n = 5; 1 independent experiment) or immunized with *Pb*WT RAS (n = 10 per time point, 1 independent experiments), and BALB/c mice, either non-immunized (n = 10; 2 independent experiments) or immunized with *Pb*WT RAS (day 6, n = 20; day 13, n = 15; and day 27, n = 20; 2 independent experiments) at the indicated days after priming. **e**, Quantification of functional luciferase activity in non-immunized (n = 5), and *Pb*WT (n = 5) or *PbSpect1p*^−^ (n = 5) RAS-immunized BALB/c mice, 48 hours after challenge with *Pb*WT SPZ. **f**, Representative flow cytometry plots (right) and frequency (left) of CD4 and CD8 T cells in the peripheral blood of *Pb*WT RAS-immunized C57BL/6 or BALB/c at the time of challenge after receiving, 4 days earlier, IgG2b (n = 10 per group) or αCD8 T cell-depleting (n = 8 per group) antibodies (2 independent experiments). Cell gating strategy shown in Extended Data Fig. 7c.**g**, **h** Representative flow cytometry plots and frequency of CD4 and CD8 T cells in (**g**) the livers and (**h**) the spleens of protected *Pb*WT RAS-immunized BALB/c mice receiving IgG2b (n = 10) or αCD8 T cell-depleting (n = 8) antibodies 4 days prior to challenge, analyzed at the end of the 12-day period after challenge (2 independent experiments). Cell gating strategy shown in Extended Data Fig. 7c. **a**-**h**, Data (two-sided Mann-Whitney test) are represented as dot plots, black line represents mean. ns – non-significant; * - p < 0.05; ** - p < 0.01; *** - p < 0.001. For complete statistical analysis, please refer to Source Data Extended Data Fig. 3. Source data

The presence of blood-stage parasites after infectious challenge of mice immunized with CT-deficient SPZ (Fig. 1) probably reflects the role of CT in promoting and/or maintaining immune mechanisms relevant for parasite clearance during hepatic infection. W-SPZ vaccination in rodents elicits both humoral and cellular responses that cooperate to eliminate SPZ and infected hepatocytes^26^. To investigate which of these protective immune mechanisms is hindered in the absence of CT, we assessed the dynamics of parasite elimination during challenge of *Pb*WT and *PbSpect1*^−^ RAS-immunized mice (Fig. 3a). At the first timepoint analysed, 8 h.p.c., both RAS-immunized cohorts showed comparable reduction in parasite liver load when compared with non-immunized controls (Fig. 3a), indicating early partial control of infection. Aligned with the notion that elevated antiplasmodial antibody titre following immunization hinder parasite establishment^27,28^, both *Pb*WT and *PbSpect1*^−^ RAS-immunized mice exhibited increased, but comparable, levels of anti-SPZ IgG before each boost and at the time of challenge (Fig. 3b). This was also the case for *PbPlp1*^−^ and *PbP36p*^−^ RAS-immunized mice (Extended Data Fig. 3a,b), which is consistent with preserved antibody responses despite impaired CT or infectivity. Assessment of the temporal dynamics of liver stage development from 16 h.p.c. with infectious SPZ showed a progressive increase in liver parasite burden in *PbSpect1*^−^ RAS-immunized mice, indicative of ongoing intrahepatic development, whereas that of *Pb*WT RAS-immunized mice remained unaltered throughout infection (Fig. 3a). Together, our data show that CT is essential for the generation of protective cellular immunity against infected hepatocytes, whereas antiplasmodial antibody responses occur independently of CT and, notably, of the extent of intrahepatic development of immunizing parasites, as indicated by the production of anti-SPZ antibodies after immunization with *PbP36p*^−^ parasites. To determine whether the requirement for CT in promoting sterile protection is specific to cellular immunity-dominated responses, we used our RAS immunization protocol (Fig. 1e) in BALB/c mice, a strain known to mount strong antibody-mediated responses^29–31^. As for C57BL/6 mice (Fig. 1b), the 15:1 *PbSpect1*^−^ to *Pb*WT inoculum ratio yielded equivalent RAS infections at 16 h.p.i. (Extended Data Fig. 3c). However, analysis of the temporal dynamics of infectious parasite elimination revealed that, unlike C57BL/6 mice (Fig. 3a), neither *Pb*WT nor *PbSpect1*^−^ RAS-immunized BALB/c mice displayed detectable liver parasite loads at any of the timepoints analysed post challenge (Fig. 3c). The rapid clearance of infectious SPZ in *Pb*WT RAS-immunized BALB/c mice correlated with the production of anti-SPZ IgG (Extended Data Fig. 3d), potentially leading to efficient SPZ neutralization before hepatocyte invasion^28^. Accordingly, no bioluminescence was detected in the livers of BALB/c mice immunized with either CT-competent or CT-deficient parasites (Fig. 3d and Extended Data Fig. 3e), with 100% of mice remaining blood-stage parasite-free over the 12-day monitoring period (Fig. 3e). Together, these data suggest that CT is dispensable when sterile protection following W-SPZ immunization relies heavily on antibody-mediated clearance of infectious parasites. To confirm that the contrasting strain-specific dependence on CT for protection (Figs. 1f–h and 3d,e) reflected a dependency on cellular versus antibody-mediated immunity, we depleted CD8 T cells in *Pb*WT RAS-immunized C57BL/6 and BALB/c mice 4 days before challenge (Fig. 3f). CD8 T cell depletion in C57BL/6 mice (Extended Data Fig. 3f) abrogated sterile protection (Fig. 3g), consistent with a dominant role for T cell-mediated clearance of infected hepatocytes^32,33^. By contrast, BALB/c mice remained blood-stage parasite-free after CD8 T cell depletion before challenge (Fig. 3h and Extended Data Fig. 3f), despite the sustained reduction in CD8 T cell frequencies in the liver and the spleen throughout the 12-day monitoring period after challenge (Extended Data Fig. 3g,h).

### CT promotes the establishment of hepatic CD8 T_RM_ T cells

Having established its critical role in eliciting protective cell-mediated immunity against *Plasmodium* liver stages (Fig. 3), we next sought to investigate how CT influenced the hepatic immune landscape in terms of cellular composition and frequency following vaccination. To this end, we used spectral flow cytometry to comprehensively profile hepatic leukocyte populations in *Pb*WT and *PbSpect1*^−^ RAS-immunized C57BL/6 mice 28 days after priming. This timepoint was selected to capture the steady-state immune milieu established after immunization and to assess CT-dependent alterations in the development or maintenance of hepatic immune populations.

**Fig. 4:**
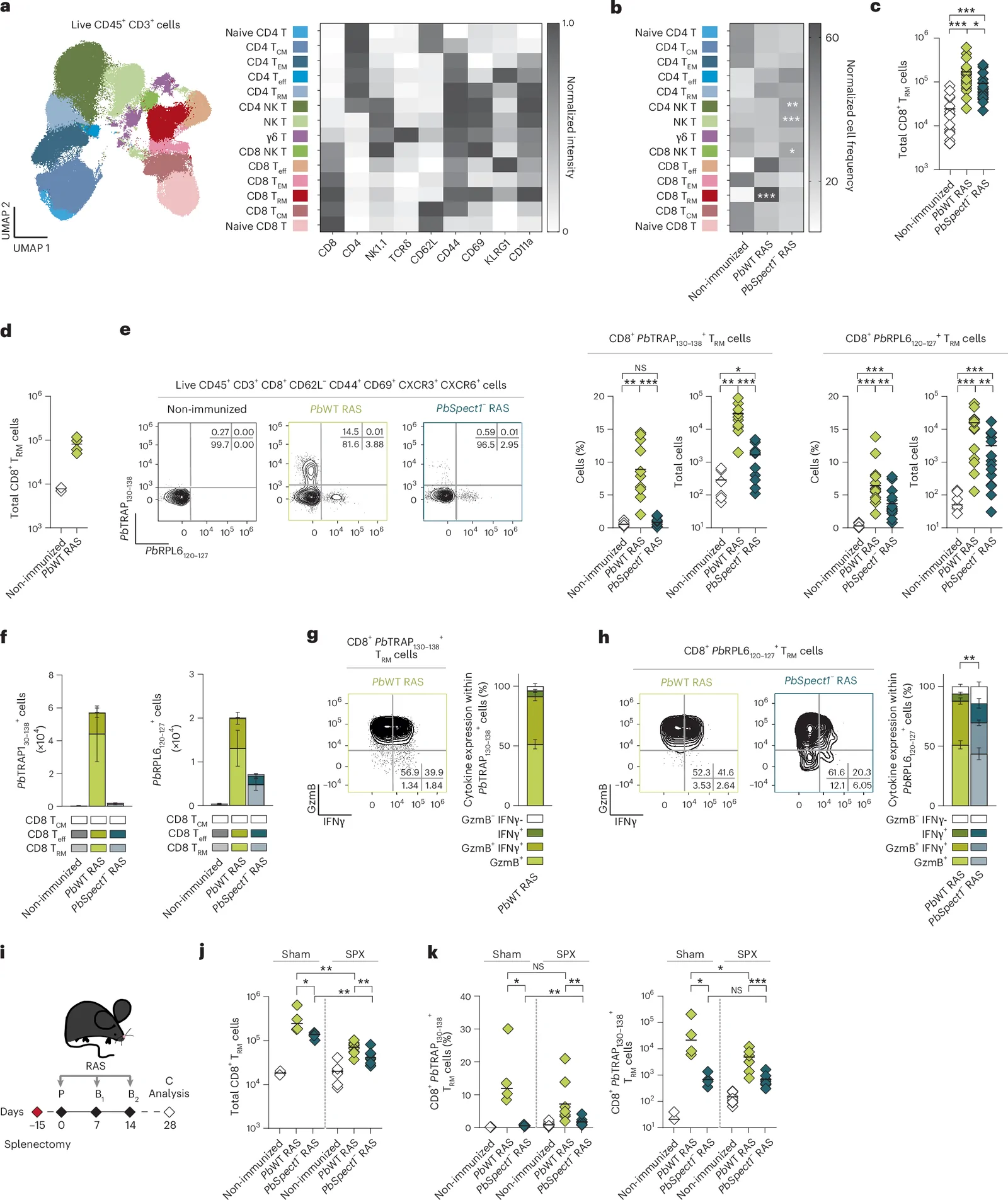
CT impacts the establishment of hepatic-resident memory CD8 T cells following W-SPZ vaccination. **a**, Uniform manifold approximation and projection (UMAP) representation (left) and mean fluorescence intensity of each hallmark marker (right) in immune cell clusters identified by FlowSOM in concatenated CD3^+^ immune cells isolated from the livers of non-infected (*n* = 6), and *Pb*WT (*n* = 10) or *PbSpect1*^−^ (*n* = 10) RAS-immunized C57BL/6J mice, 28 days after prime (two independent experiments). Cell gating strategy is shown in Extended Data Fig. 6a. **b**, Heat map of normalized cell frequencies within each identified cluster of CD3^+^ lymphocytes in the livers of non-infected, and *Pb*WT or *PbSpect1*^−^ RAS-immunized C57BL/6J mice, 28 days after prime. **c**, Quantification of CD69^+^ KLRG1^−^ T_RM_ CD8 T cells in the livers of non-immunized (*n* = 15), and *Pb*WT (*n* = 24) or *PbSpect1*^−^ (*n* = 25) RAS-immunized C57BL/6J mice, 28 days after prime (five independent experiments). Cell gating strategy is shown in Extended Data Fig. 6b. **d**, Quantification of CD8 T_RM_ cells in the livers of non-immunized (*n* = 3) and *Pb*WT (*n* = 4) RAS-immunized C57BL/6J mice, 75 days after prime (one experiment). **e**, Representative flow cytometry plots and frequency and quantification of *Pb*TRAP_130–138_-specific and *Pb*RPL6_120–127_-specific CD8 T_RM_ cells in the livers of non-immunized (*n* = 6), and *Pb*WT (*n* = 10) or *PbSpect1*^−^ (*n* = 10) RAS-immunized C57BL/6J mice, 28 days after prime (two independent experiments). Cell gating strategy is shown in Extended Data Fig. 6b. **f**, Distribution of *Pb*TRAP_130–138_-specific (left) and *Pb*RPL6_120–127_-specific (right) CD8 T cells in circulating (central memory T (T_CM_) cell and effector T (T_eff_) cell) and resident (T_RM_) cellular compartments in the livers of non-immunized (*n* = 9), and *Pb*WT (*n* = 14) or *PbSpect1*^−^ (*n* = 14) RAS-immunized C57BL/6J mice 28 days after prime (three independent experiments). **g**,**h**, Representative flow cytometry plots and frequency of GzmB and IFNγ expression in *Pb*TRAP_130–138_-specific CD8 T_RM_ cells in the livers of *Pb*WT (*n* = 10) RAS-immunized C57BL/6J mice (**g**) and *Pb*RPL6_120–127_-specific CD8 T_RM_ cells in the livers of *Pb*WT (*n* = 10) or *PbSpect1*^−^ (*n* = 10) RAS-immunized C57BL/6J mice (**h**) 28 days after prime (two independent experiments). Cell gating strategy is shown in Extended Data Fig. 6b. **i**, Protocol of splenectomy and RAS immunizations. **j**, Quantification of T_RM_ CD8 T cells in the livers of non-immunized, and *Pb*WT or *PbSpect1*^−^ RAS-immunized C57BL/6J mice, either sham-operated (*n* = 3, *n* = 4 and *n* = 5, respectively; one independent experiment) or SPX (*n* = 9, *n* = 8 and *n* = 9, respectively; two independent experiments), 28 days after prime. Cell gating strategy is shown in Extended Data Fig. 6b. **k**, Frequency and quantification of *Pb*TRAP_130–138_-specific CD8 T_RM_ cells in the livers of non-immunized, and *Pb*WT or *PbSpect1*^−^ RAS-immunized C57BL/6J mice, either sham-operated (*n* = 3, *n* = 4 and *n* = 5, respectively; one independent experiment) or SPX (*n* = 9/10, *n* = 8 and *n* = 9, respectively; two independent experiments), 28 days after prime. Cell gating strategy is shown in Extended Data Fig. 6b. In **c**–**e**,**j**,**k**, data are represented as dot plots, with the black line representing the mean; in **f**–**h**, data are mean ± s.e.m.  *P < 0.05, **P < 0.01, ***P < 0.001, two-sided Mann–Whitney test (**b**–**g**,**j**,**k**) and two-way repeated measures ANOVA (**h**). T_EM_, effector memory T cell. Source data

**Extended Data Fig. 4:**
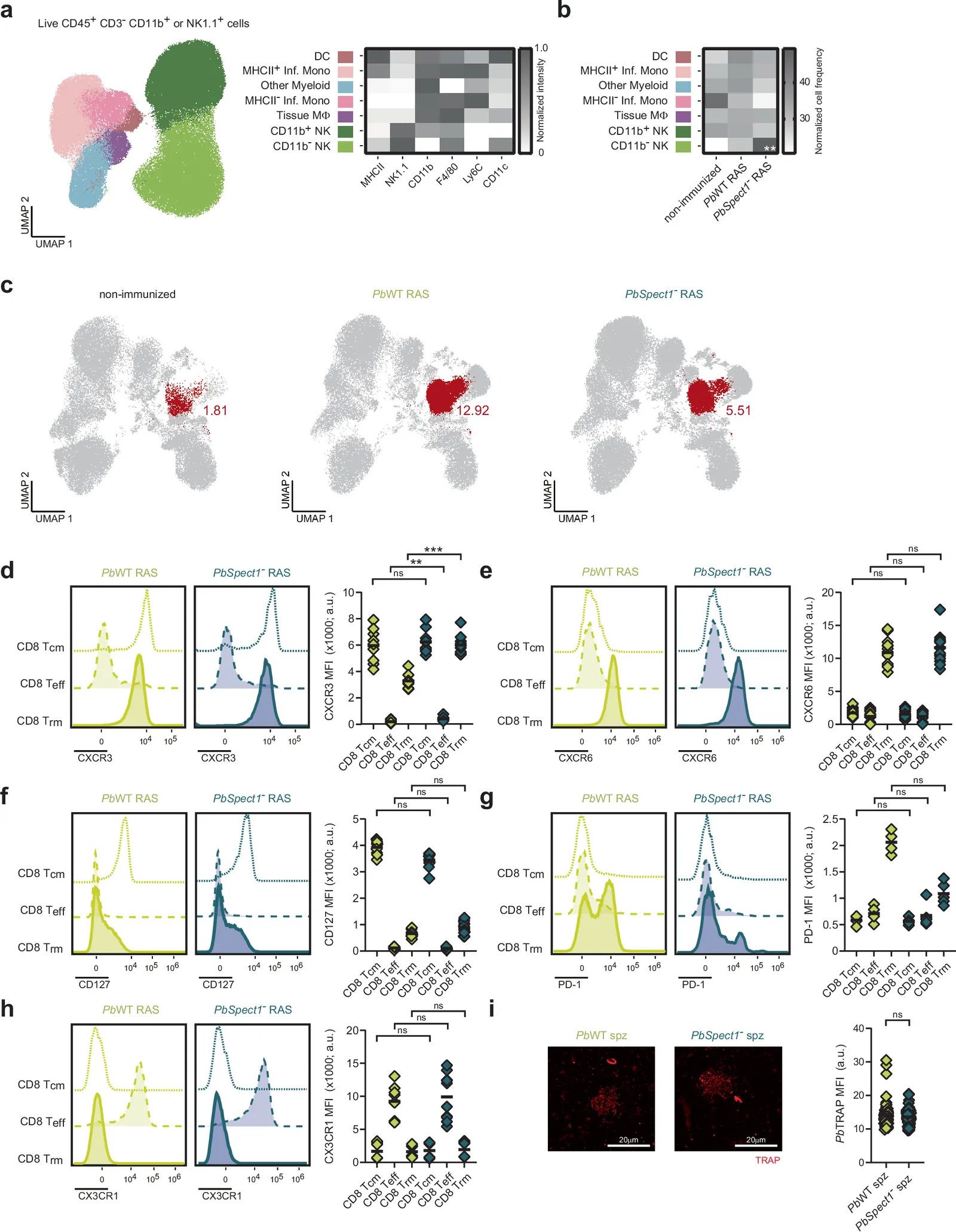
Innate and CD8 Trm immune responses following W-SPZ vaccination. **a**, Uniform manifold approximation and projection (UMAP) representation (left) and mean fluorescence intensity (MFI) of each hallmark marker (right) in immune cell clusters identified by FlowSOM in concatenated CD3^−^ immune cells expressing CD11b and/or NK1.1 isolated from the livers of non-infected (n = 5), and *Pb*WT (n = 9) or *PbSpect1*^−^ (n = 9) RAS-immunized C57BL/6 J mice, 28 days after prime (2 independent experiments). DC – dendritic cell; Inf. Mono – inflammatory monocyte; MΦ – macrophage; NK – natural killer. Cell gating strategy shown in Extended Data Fig. 6a. **b**, Heatmap of normalized cell frequencies within each identified cluster of CD3⁻ immune cells expressing CD11b and/or NK1.1 in the livers of non-infected, and *Pb*WT or *PbSpect1*^−^ RAS-immunized C57BL/6 J mice, 28 days after prime. **c**, Uniform manifold approximation and projection (UMAP) representation of concatenated CD3^+^ immune cells isolated from the livers of non-infected (n = 6) (left), and *Pb*WT (n = 10) (middle) or *PbSpect*1^−^ (n = 10) (right) RAS-immunized C57BL/6 J mice, 28 days after prime (2 independent experiments). Frequencies of the highlighted CD8 T_rm_ cell cluster (red) are shown for each condition. **d-h**, Representative histograms (left) and quantification (right) of mean fluorescence intensity (MFI) of (**d**) CXCR3 (n = 9 and n = 10, respectively; 2 independent experiments), (**e**) CXCR6 (n = 14 and n = 15, respectively; 3 independent experiments), (**f**) CD127 (n = 9 and n = 10, respectively; 2 independent experiments), (**g**) PD-1 (n = 9 and n = 10, respectively; 2 independent experiments) and (**h**) CX3CR1 (n = 9 and n = 10, respectively; 2 independent experiments) surface expression in CD8 T_cm_, T_eff_ and T_rm_ cells in the livers of *Pb*WT or *PbSpect1*^−^ RAS-immunized C57BL/6 J mice 28 days after prime. Cell gating strategy shown in Extended Data Fig. 6b. **i**, Representative fluorescence confocal microscopy images (left) and quantification (right) of mean fluorescence intensity (MFI) of TRAP in the trails of *Pb*WT (n = 69) or *PbSpect1*^−^ (n = 75) SPZ during *in vitro* gliding motility assays over a 30-minute period (2 independent experiments). Images were acquired with 40x magnification and scale bar represents 20 µm. **b**, Data (two-sided Mann-Whitney test) are represented as a heatmap. **d-i**, Data (two-sided Mann-Whitney test) are represented as dot plots, black line represents mean. ns – non-significant; * - p < 0.05; ** - p < 0.01; *** - p < 0.001. For complete statistical analysis, please refer to Source Data Extended Data Fig. 4. Source data

Hierarchical, unsupervised clustering of hallmark markers using FlowSOM on concatenated CD3^+^ lymphocytes and CD3^−^ cells expressing CD11b and/or NK1.1 identified 14 and 7 distinct cell clusters, respectively (Fig. 4a and Extended Data Fig. 4a). Of these, *PbSpect1*⁻ RAS-immunized mice exhibited increased frequencies of natural killer (NK) T cell clusters and of hepatic CD11b^−^ NK1.1^+^ cells compared with mice immunized with *Pb*WT RAS (Fig. 4b and Extended Data Fig. 4b). NK and NK T cells have previously been associated with the elimination of *Plasmodium* liver stages^34,35^, arguing against a causal link between the expansion of these cell clusters and the loss of protection observed following *PbSpect1*⁻ RAS immunizations (Fig. 1). By contrast, only one cluster showed altered abundance associated with protection. CD69^+^ KLRG1⁻ CD8 tissue-resident memory T (T_RM_) cells were enriched, in both frequency and total numbers, in protected mice immunized with *Pb*WT RAS, but significantly under-represented in the unprotected *PbSpect1*^−^ RAS-immunized cohort (Fig. 4b,c and Extended Data Fig. 4c). Targeted phenotypic characterization confirmed hallmark features of hepatic tissue residency^32^, including high expression of CXCR3 and CXCR6, accompanied by increased CD127 and PD-1 levels (Extended Data Fig. 4d–g), which is consistent with long-lived, antigen-experienced resident cells^36,37^. The absence of CX3CR1 expression (Extended Data Fig. 4h) further supported exclusion from a circulating effector phenotype^38^. Reflecting the long-term persistence of memory T cell subsets, hepatic CD8 T_RM_ cells in the *Pb*WT-immunized cohort were stably maintained at 75 days post priming (Fig. 4d), a timepoint associated with substantial protection against SPZ challenge (Fig. 2d). Together, these findings show that lack of CT by immunizing parasites impairs the establishment of bona fide hepatic CD8 T_RM_ cells, a population required for localized and durable immunity against liver stage parasites following W-SPZ vaccination^32,39^.

Repetitive exposure to RAS generates long-lived *Plasmodium*-specific CD8 T_RM_ cell populations that persist within the hepatic niche independently of circulating memory pools and are essential for sterile protection^39–41^. Therefore, we next assessed whether the CT-dependent differences observed in the overall hepatic CD8 T_RM_ cell compartment extended to the generation of *Plasmodium*-specific CD8 T_RM_ cells. For this, we focused on two well-defined *Plasmodium* epitopes with distinct expression patterns: the *thrombospondin-related anonymous protein* (*Pb*TRAP_130–138_)^40^, which is expressed mostly at the SPZ stage and shed during parasite locomotion^42,43^, and the putative 60S *ribosomal protein L6* (*Pb*RPL6_120–127_)^44^, associated with intrahepatic parasite development^45^. When compared with non-immunized controls, *Pb*WT RAS-immunized C57BL/6 mice displayed increased frequencies and absolute numbers of CD8 T_RM_ cells specific for both epitopes 28 days after priming (Fig. 4e). By contrast, *Pb*TRAP_130–138_-specific CD8 T_RM_ cells were markedly reduced in the livers of *PbSpect1*⁻ RAS-immunized mice, whereas *Pb*RPL6_120–127_-specific CD8 T_RM_ cells remained detectable, albeit with reduced abundance (Fig. 4e). *Plasmodium*-specific CD8 T_RM_ cell hepatic subsets in both CT-competent and CT-deficient RAS-immunized mice were preferentially enriched within the tissue-resident compartment (Fig. 4f) and exhibited a cytotoxic profile characterized by increased production of granzyme B (GzmB) and interferon-γ (IFNγ) following ex vivo stimulation, although the distribution of these effector molecules differed between *Pb*RPL6_120–127_-specific cells from *Pb*WT- and *PbSpect1*⁻-immunized mice (Fig. 4g,h). Together, these observations indicate that the absence of CT during immunization does not uniformly impair CD8 T_RM_ cell formation, but instead selectively compromises the establishment of *Pb*TRAP_130–138_-specific CD8 T_RM_ cells, reflecting a CT-dependent requirement for responses directed against antigens highly expressed at the SPZ stage^46^. Such reduction was not associated, however, with diminished levels of TRAP shed during gliding motility by *PbSpect1*⁻ SPZ compared with their wild-type counterparts (Extended Data Fig. 4i).

**Extended Data Fig. 5:**
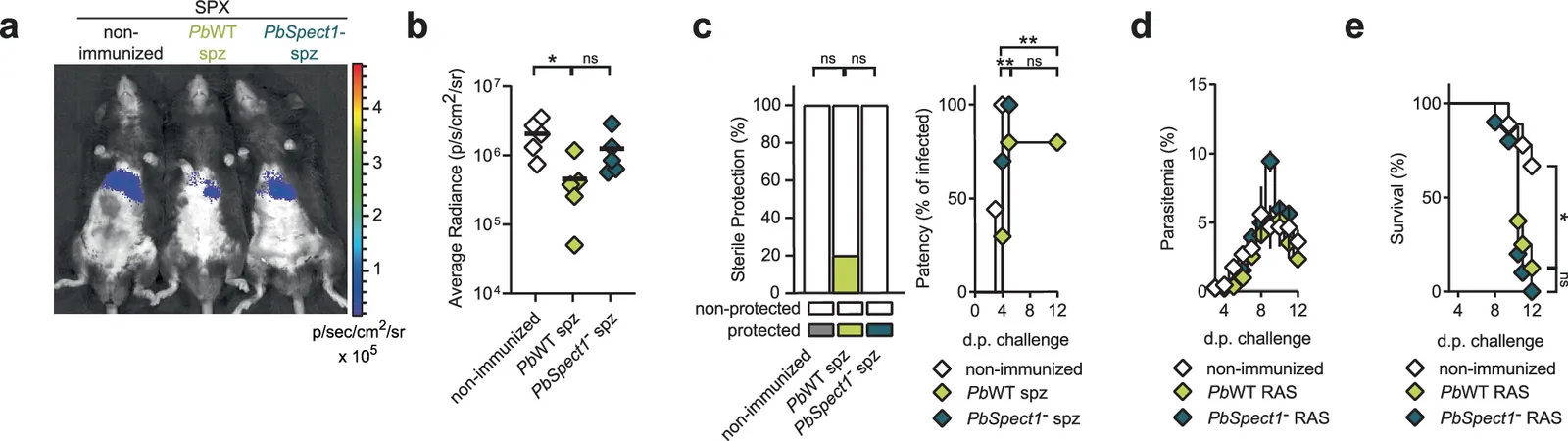
Sterile protection following W-SPZ vaccination is lost in splenectomized mice. **a**, **b** Representative luciferase bioluminescence image (**a**) and quantification of functional luciferase activity (**b**) of non-immunized (n = 5), and *Pb*WT (n = 5) or *PbSpect1*^−^ (n = 5) RAS-immunized splenectomized (SPX) C57BL/6 J mice, 48 hours after challenge with *Pb*WT SPZ. **c**, Sterile protection, represented as percentage of blood stage parasite-free mice (left), and time to patency (right) of non-immunized (n = 9), and *Pb*WT (n = 10) or *PbSpect1*^−^ (n = 10) RAS-immunized asplenic C57BL/6 mice in the 12-days period following challenge with *Pb*WT SPZ (2 independent experiments). **d, e** Parasitemia (**d**) and survival (**e**) in non-immunized (n = 9), and patent *Pb*WT (n = 10) or *PbSpect1*^−^ (n = 10) RAS-immunized asplenic C57BL/6 mice in the 12-days period following challenge with *Pb*WT SPZ (2 independent experiments). Parasitemia gating strategy shown in Extended Data Fig. 7a. **b**, Data (Mann-Whitney test) are represented as dot plots, black line represents mean. **c**, left panel, Data (Mann-Whitney test) are represented as bars ± SEM. **c**, right panels, Data (Mann-Whitney test) are represented as the mean day to patency. **d**, Data (Mann-Whitney test) are represented as mean percentage of infected erythrocytes ± SEM. **e**, Data (Log rank (Mantel-Cox) test) are represented as mean survival rate. ns – non-significant; * - p < 0.05; ** - p < 0.01; *** - p < 0.001. For complete statistical analysis, please refer to Source Data Extended Data Fig. 5. Source data

Given that CD8 T cell responses following RAS immunization have been shown to initiate in the spleen^41^, we next asked whether the CT-dependent impairment in *Plasmodium*-specific CD8 T_RM_ cell formation reflected altered splenic priming by performing RAS immunizations in splenectomized (SPX) C57BL/6 mice (Fig. 4i). Removal of the spleen resulted in a global reduction in the frequencies of hepatic CD8 T_RM_ cells independently of the CT competency of immunizing parasites (Fig. 4j). Notably, however, the relative reduction in *Pb*TRAP_130–138_-specific CD8 T_RM_ cells observed in mice immunized with CT-deficient *PbSpect1*⁻ RAS was preserved in SPX hosts, when compared with the *Pb*WT RAS-immunized cohort (Fig. 4k). The overall reduction in hepatic CD8 T_RM_ cells following splenectomy was associated with loss of sterile protection across all SPX cohorts, with 80% and 100% of mice immunized with CT-competent or CT-deficient RAS becoming patent on days 4.6 ± 0.2 and 4.3 ± 0.1, respectively (Extended Data Fig. 5a–e). Together, these observations suggest that while splenic priming contributes to the overall magnitude and protective capacity of cellular immune response following RAS exposure, CT at the site of infection is important for shaping the antigen-specific composition of the hepatic CD8 T_RM_ cell pool.

## Discussion

Despite major advances in our understanding of host immune responses during *Plasmodium* liver infection, parasite-intrinsic determinants that shape these responses remain poorly defined. Efforts in vaccine development have mostly focused on identifying and targeting specific antigens or epitopes capable of eliciting protective immune responses. Our findings challenge this antigen-centric view by demonstrating that parasite behaviour traits are a critical contributor to cellular immunity elicited during W-SPZ immunization.

In this study, we showed that CT by immunizing SPZ, which occurs before hepatocyte invasion^4^, was a major immunogenic determinant for inducing sterilizing immunity following W-SPZ vaccination. This finding contrasts with the prevailing view that vaccine-induced protection is primarily driven by productive hepatocyte infection and by the extent of intrahepatic parasite development^14^. Using multiple genetic parasite models, we showed that even when CT-deficient parasites were allowed to fully replicate in hepatocytes, they failed to confer protection. Conversely, CT-competent parasites that are replication-impaired still elicited robust protection, equivalent to wild-type parasites. These data establish CT as a key immunogenic event and demonstrate that it is uncoupled from sterilizing protection following W-SPZ vaccination from productive *Plasmodium* intrahepatic development.

RAS-based immunizations elicit both humoral and cellular responses, each contributing to preclude re-infection of immunized hosts^32,47^. The observation that sterile protection was abrogated in C57BL/6 mice immunized with CT-deficient parasites, despite high SPZ antibody levels, underscores that CT contributes to cell-mediated, but not antibody-mediated, immunity. Consistent with this and aligning with observations by others^48^, CD8 T cell depletion in CT-competent RAS-immunized C57BL/6 mice abrogated protection. By contrast, in BALB/c mice, which favour antibody-mediated immunity^29–31^, both CT-competent and CT-deficient immunizing parasites elicited comparable levels of protection, independent of CD8 T cells. These results highlight how host genetics dictate immune correlates of protection, and underscore CT as a non-redundant component for inducing CD8 T cell-mediated immunity in hosts where this response dominates.

Cellular responses, particularly those dependent on CD8 T cells, are essential for the clearance of infected hepatocytes^48^, with prolonged protection following malaria vaccination being associated with the presence of liver-resident CD8 T_RM_ cells^32^. Using unsupervised clustering of liver leukocytes, we found that CD8 T_RM_ cells expanded significantly following immunization with CT-competent versus CT-deficient RAS. Within this compartment, *Pb*TRAP_130–138_-specific CD8 T_RM_ cells were markedly under-represented in mice immunized with CT-deficient RAS, revealing that this parasite behaviour trait shapes the antigenic composition of the hepatic CD8 T_RM_ cell pool. Such selective loss is particularly notable since repeated RAS immunization has been shown to skew the hepatic CD8 T_rm_ compartment towards specificities targeting highly abundant SPZ antigens, including TRAP, which become dominant contributors to protection^39^. Our findings provide a mechanistic explanation for this bias, suggesting that by facilitating antigen dissemination across multiple host cells early during infection, CT may preferentially amplify cellular responses against SPZ-derived antigens, and thereby shape both the composition and protective capacity of the hepatic CD8 T_RM_ cell compartment. Consistent with this model, complementary work by Pohl et al.^49^ demonstrates that the absence of CT prevents activation of bone marrow-derived macrophages and consequently impairs the priming of protective CD8 T cell responses, revealing an innate immune mechanism that may account for the downstream defects in hepatic CD8 T_RM_ cell abundance and specificity observed following immunization with CT-deficient parasites.

While it may seem paradoxical that *Plasmodium* parasites evolved a behaviour that promotes CD8 T cell responses capable of killing infected hepatocytes, this probably represents an evolutionary compromise. In infections occurring in endemic settings, this trait is essential for parasite infectivity, allowing SPZ to cross host barriers in the skin and at the liver sinusoids^50,51^. In such case, the number of SPZ that successfully reach the liver upon mosquito transmission is relatively low^52^, contrasting with the artificially high parasite loads used in W-SPZ vaccination regimens in both rodents^53^ and humans^10^. Therefore, it is likely that, under natural transmission conditions, the immunostimulatory effects of CT by *Plasmodium* parasites reflect an evolutionary trade-off to ensure successful infection in endemic populations, whereas during vaccination, the amplified exposure to CT-dependent immunogenic cues drives robust immune activation. These findings carry important implications for the development of malaria vaccines for human use.

Traditionally, vaccine design has prioritized the search for optimal antigens, epitopes and immune signatures. While this approach remains essential, our findings linking CT and protective immunity should broaden the focus from solely considering the antigenic content of immunizing strategies to include the dynamic interactions between the parasite and host tissues. This conceptual shift opens new avenues for malaria vaccine development, where parasite behaviour can be harnessed to shape effective and durable immunity. Responses to W-SPZ vaccines in humans are known to be heterogeneous, with a fraction of vaccinees failing to develop sterile protection^10,11^. The reasons behind such variability are not fully understood but may include differences in vaccine delivery, host genetics and immune history, as well as limitations in directly interrogating protective immune mechanisms in humans. As a result, while mechanistic insight into cellular immunity can be obtained in animal models, its contribution to protection in humans remains largely correlative, in part because key effector populations such as liver-resident CD8 T cells are not readily accessible for direct assessment. Our data raise the possibility that parasite behaviour during vaccination, including its capacity to traverse host cells, represents a previously unappreciated determinant of vaccine outcome that may influence how different immune effector mechanisms are engaged and balanced across individuals. Next-generation W-SPZ vaccines may benefit from engineering or selecting immunizing parasites with optimized behavioural properties, particularly, enhanced CT motility, to promote effective and long-lasting CD8 T_RM_ cell responses. Moreover, the currently licensed malaria vaccines RTS,S and R21, consisting of subunit formulations targeting the CSP of *Plasmodium* *falciparum*, rely heavily on humoral immunity^54^ and consistently fail to prevent infection, albeit reducing clinical disease burden^6–9^. The identification of molecular and inflammatory cues induced by CT may enable the design of adjuvants that replicate its immunostimulatory effect in cellular immunity, extending the applicability of this principle beyond W-SPZ vaccines.

## Methods

### Mice

C57BL/6 and BALB/c wild-type mice were obtained from Charles River Laboratories. All mice in this study were housed in specific pathogen-free conditions, at 23 °C and 40–50% humidity with a 12-h light/12-h dark cycle, in accordance with the European Commission recommendations on housing and care of animals. For immunizations and infections, both male and female mice (5–6 weeks old) were used. Animal experimentation protocols were approved by the institutional animal welfare body, ORBEA-GIMM, and by the Direcção-Geral de Alimentação e Veterinária (license no. 002469/2021). All procedures were performed in strict accordance with the Directive 2010/63/EU for the care and use of animals for scientific purposes.

### Parasites

For this study, *Anopheles stephensi* female mosquitoes, bred and housed at GIMM Insectary, were infected with the following *Pb* ANKA (*Pb*ANKA) parasite lines: (1) *Pb*ANKA expressing a GFP–luciferase fusion protein (676m1cl1; RMgm-29)^55^, herein referred to as *Pb*WT, (2) marker-free *Pb*ANKA expressing GFP (2.34)^56^, herein referred to as *Pb*GFP, (3) *Pb*ANKA genetically deleted for the *Sporozoite microneme protein essential for cell traversal 1* (*Spect1*) gene (RMgm-138)^18^, herein referred to as *PbSpect1*^−^, (4) *Pb*ANKA genetically deleted for the *P36p* gene (417m0cl2; RMgm-41)^25^, herein referred to as *PbP36p*^−^ and (5) *Pb*ANKA genetically deleted for the *perforin-like protein 1* (*Plp1*^−^) gene, generated in this study and herein referred to as *PbPlp1*^−^. The generation of *PbPlp1*^−^ was carried out using the *Pb*GFP parasite line and the *Pb*GEM-293340 (PlasmoGEM) vector. *Pb*GEM-293440 contains a modified locus of the PBANKA_100630 gene (*Plp1*; PlasmoDB), consisting of the *Pbeef1aa* promoter, followed by the human dihydrofolate reductase (*hDHFR*) gene, and the bifunctional yeast enzyme cytosine deaminase and uridyl phosphoribosyl transferase (*yFCU*). The modified target locus was integrated in the *Pb*GFP parasite line by double crossover homologous recombination, recombinant parasites were selected with pyrimethamine and dilution cloning was carried out as previously described^55^. The genomic DNA (gDNA) of parasite clones was isolated from parasitized blood using the NZY Blood gDNA Isolation kit (NZYtech), and genotyped by PCR using NZYTaq II 2× Colourless Master Mix (0.1 U µl^−1^; NZYtech,) and primers specific for the *Plp1* WT locus (5′ primer P2 (ACCGTTAGATCTGATTGCGGCCA) and 3′ primer P4 (ACACATAGCGAAACCATGTTGTCT)) and for the correct integration of the modified target locus (5′ primer P1 (TGCGAACTGAGATACGCAGGGA) and 3′ primer P3 (CATACTAGCCATTTTATGTG)). For this, 1 µg of total gDNA was used to amplify the WT and integration loci in a T100 Thermal Cycler (Bio-Rad), with a PCR programme consisting of 1 cycle at 95 °C for 5 min, followed by 40 cycles of 95 °C for 30 s, 50 °C for 30 s and 68 °C for 90 s, and a final cycle of 68 °C for 10 min. The resulting PCR products were resolved in a 1% agarose gel and visualized in an Amersham Imager 680. Clones were considered ablated for the *Plp1* gene by the presence of the amplification product corresponding to the correct integration of the modified target locus and absence of the WT locus.

### Immunizations and infections

SPZ were obtained by the dissection of salivary glands of infected mosquitoes from day 20 post blood meal onwards. All administrations of infectious or attenuated SPZ were carried out in 200 µl of DMEM (Gibco) via retro-orbital route in mice lightly anaesthetized with isoflurane (isoflurane 1,000 mg ml^−1^; Iso-Vet). RAS-based immunizations consisted of three administrations, on a weekly basis, of 10^4^ *Pb*WT, 15 × 10^4^ *PbSpect1*^−^, 15 × 10^4^ *PbPlp1*^−^ or 10^4^ *PbP36p*^−^ γ-irradiated SPZ (16,000 rads; Caesium137 γ-source, GammaCell 3000 Elan, Thetratronics). Non-immunized controls received equivalent amounts of salivary gland extract from uninfected *A. stephensi* mosquitoes using the same volume and administration route. CPS-based immunizations consisted in three administrations, on a weekly basis, of 10^4^ *Pb*WT or 15 × 10^4^ *PbSpect1*^−^ infectious SPZ. Mice were administered azithromycin (16 mg ml^−1^; Farmoz) in 200 µl of saline (NaCl 0.9%; Braun), via intraperitoneal injection, on days 0, 1 and 2 post each immunization (CPS-AZ), or were given continuous access to water supplemented with chloroquine diphosphate salt (0.288 mg ml^−1^; Sigma) and glucose (15 g l^−1^; NZYtech), from days 1 to 6 after each immunization (CPS-CQ). Non-immunized controls received equivalent amounts of salivary gland extract from uninfected *A. stephensi* mosquitoes using the same volume and administration route, and were exposed to azithromycin or chloroquine in doses equivalent to those of immunized cohorts. Mice from all immunization regimens and respective controls were challenged with 3 × 10^4^ *Pb*WT infectious SPZ, 28 days post priming.

### Splenectomy

C57BL/6 mice were anaesthetized with 2–3% isoflurane (isoflurane 1,000 mg ml^−1^; Iso-Vet) before receiving buprenorphine (1.5 μg ml^−1^) pre-emptive analgesia, and were prepared for surgery by shaving and disinfecting the dorsal left thoracolumbar region. The abdominal cavity was opened by creating an incision left of the spine. The spleen was exposed, and the splenic vessels were isolated with a hemostat and cauterized, before being cut distally to the cauterized section, enabling the removal of the spleen. The abdomen and the skin were closed with sutures and surgical glue (3 M Vetbond), and the animals were monitored for pain and given continuous access to water supplemented with buprenorphine (0.9 μl ml^−1^) for 3 days post surgery. All immunization protocols were initiated 15 days after splenectomy.

### In vivo bioluminescence

Liver parasite burden at 48 h.p.c. was assessed by in vivo functional luciferase activity using the IVIS Lumina imaging system (PerkinElmer) and Living Image 3.0 software. In brief, mice were administered d-luciferin (180 mg kg^−1^ of body weight; PerkinElmer), diluted in 300 μl of PBS, 5 min before being deeply anaesthetized with a ketamine (100 mg kg^−1^ of body weight, Ketamidor; Richter Pharma) and xylazine (10 mg kg^−1^ of body weight, Rompum; Bayer) mixture via intraperitoneal injection. After 5 min, images were acquired with a 12.5-cm field of view, medium binning factor and exposure time of 3 min. Quantitative analysis of bioluminescence in all experimental cohorts was performed by measuring the luminescence signal radiance using Living Image 3.0 software.

The day of patency of blood-stage infection was determined by detecting the presence of bioluminescence activity of circulating intraerythrocytic parasites using the Firefly Luciferase Assay kit 2.0 (Biotum) in a previously established blood bioluminescence assay^57^. In brief, 5 µl of blood was collected from days 3 to 12 after challenge from the tail vein of each mouse into 45 µl of lysis buffer 1×, and kept at −20 °C until analysis. Bioluminescence was detected by adding 50 µl of d-luciferin, previously diluted in Firefly Luciferase Assay Buffer (0.2 mg ml^−1^; Biotum), to 15 µl of blood sample lysate, immediately measuring emitted luminescence, thrice, with an integration time of 100 ms, using a Spark multimode microplate reader (Tecan). Blood-stage infection was considered patent when luminescence values increased above 100 for two consecutive days.

### ECM assessment

Mouse survival and ECM establishment were evaluated twice daily, from day 6 post challenge onwards, by the emergence of neurological symptoms such as ataxia and paralysis, assessed by the ability of mice to self-right.

### SPZ gliding motility and TRAP protein shedding assessment

Assessment of gliding motility of *Pb* SPZ was carried out as previously described^58^. In brief, 25,000 salivary gland SPZ were dissected from infected *Anopheles* mosquitoes as described above, and incubated in complete DMEM medium for 30 min at 37 °C, on top of glass coverslips. After incubation, SPZ were fixed with 4% paraformaldehyde (PFA; Santa Cruz Biotechnology), and stained with mouse anti-*Pb*CSP (3D11) or rabbit anti-*Pb*TRAP^43^ primary antibodies followed by incubation with Alexa Fluor 568-conjugated donkey anti-rabbit IgG (4 μg ml^−1^; Invitrogen) and Alexa Fluor 647-conjugated donkey anti-mouse IgG-conjugated (4 μg ml^−1^; Invitrogen) antibodies. Coverslips were imaged using an LSM 980.32 with Airyscan microscope (Zeiss) at 40×, and SPZ gliding motility was quantified by counting the number of CSP circles formed by each individual SPZ.

Shedding of TRAP by *Pb* SPZ during gliding was assessed by measuring the TRAP-specific fluorescence intensity from immunofluorescence images of gliding trails. Images were analysed using a macro that quantified the mean fluorescence intensity within regions of interest drawn around gliding trails. A fluorescence background was established in gliding trails stained with Alexa Fluor 568-conjugated donkey anti-rabbit IgG (4 μg ml^−1^; Invitrogen) in the absence of anti-*Pb*TRAP primary antibody. Only pixels with an intensity above background were quantified.

### SPZ intrahepatic development and cell wounding assessment by immunofluorescence

In vitro infections were performed in HepG2 cells seeded on coverslips (12 mm; Menzel-Gläser) at a density of 100,000 cells per well of a 24-well plate (Costar), cultured in DMEM medium supplemented with 10% fetal bovine serum, 2 mM glutamine and 100 U ml^−1^ penicillin–streptomycin (DMEMC) (all Gibco), and maintained at 37 °C and 5% CO_2_ 24 h before infection. For the assessment of cell wounding, rhodamine-labelled Dextran (0.5 mg ml^−1^; 10,000 Da, lysine fixable; Molecular Probes) was added to each well immediately before infection with a 1:1 ratio of cells to *Pb*WT or *PbPlp1*^−^ SPZ. Plates were incubated at 37 °C and 5% CO_2_, and infection was halted 2 h.p.i. For assessment of in vitro intrahepatic development, seeded HepG2 cells were infected with *Pb*WT or *Pbplp1*^−^ SPZ as before, and infection was halted 48 h.p.i. All cells were fixed with 4% PFA (Santa Cruz Biotechnology).

In vivo infections for assessment of *Plasmodium*’*s* intrahepatic development were carried out as previously described. Then, 42 h.p.i., mice were killed by CO_2_ narcosis, and the livers were collected and fixed in 4% PFA overnight at 4 °C. The livers were equilibrated in a 30% sucrose solution for 48 h before embedding in optimal cutting temperature compound (Sakura Finetek), and frozen at −80 °C. Tissue sections were serially cut in 10-µm-thick slices in a CM2050S cryostat (Leica) and mounted in glass slides (Marienfeld).

Fixed HepG2 cells were permeabilized with 0.1% (vol/vol) Triton X-100 (Sigma) before incubation with a blocking solution of 1% (wt/vol) of Albumin Bovine Fraction V (BSA) (NZYtech). The thawed slides with liver sections were permeabilized and blocked with 5% BSA (wt/vol) (NZYtech) 0.3% (vol/vol) Triton X-100 (Sigma) in PBS.

For cell wounding assays, cells were incubated with Alexa Fluor 488-conjugated rabbit polyclonal anti-GFP antibody (4 μg ml^−1^; Invitrogen) and Hoechst 33342 (0.01 μg ml^−1^; Invitrogen), all diluted in blocking solution, for 90 min at room temperature. For in vitro and in vivo assessment of *Plasmodium*’*s* intrahepatic development, samples were incubated overnight at 4 °C with goat anti-UIS4 polyclonal antibody (10 μg ml^−1^; Sicgen) followed by incubation with Alexa Fluor 568-conjugated donkey anti-goat IgG (4 μg ml^−1^; Invitrogen), Alexa Fluor 488-conjugated rabbit anti-GFP polyclonal antibody (4 μg ml^−1^; Invitrogen) and Hoechst 33342 (0.01 μg ml^−1^; Invitrogen). For in vivo immunofluorescence staining, all antibodies were diluted in 5% BSA (wt/vol) (NZYtech) 0.3% (vol/vol) Triton X-100 (Sigma) in PBS. For all immunofluorescence assays, samples were mounted in Fluoromount G (Invitrogen) on microscope slides (Marienfeld). Mounted slides were allowed to dry in the dark at 4 °C for at least 24 h before imaging. Images were acquired using an LSM 880 with Airyscan microscope (Zeiss) at 20× (in vivo intrahepatic development), or at 63× (in vitro assays) magnification. EEF size was assessed using a macro in Fiji Image J (NIH).

### Isolation of hepatocytes for in vivo cell wounding assessment

C57BL/6 mice received 160 μg of rhodamine-labelled Dextran (10,000 Da, lysine fixable; Molecular Probes) diluted in PBS immediately before inoculation of 5 × 10^5^ *Pb*WT or 5 × 10^5^ *PbP36p*^−^ infectious SPZ, both via retro-oribtal route. Then, 2 h after, hepatocytes were isolated by a modified two-step collagenase perfusion method^59^. In brief, livers were perfused in situ through the vena cava, cutting the portal vein to enable outflow, with 15 ml of perfusion buffer (HBSS no Ca^2^^+^, no Mg^2^^+^ and no phenol red (Gibco), supplemented with EDTA (0.5 M; Gibco), and HEPES (25 mM; Gibco)) pre-warmed at 42 °C. Subsequently, the livers were perfused with 25 ml of digestion buffer (HBSS with Ca^2^^+^, Mg^2^^+^ and phenol red (Gibco), supplemented with HEPES (25 mM; Gibco) and Liberase Blendzyme 3 recombinant collagenase (0.04 mg ml^−1^; Roche Diagnostics)) pre-warmed at 42 °C. After perfusion, liver tissue was gently dispersed in a single-cell suspension using a pair of forceps. Single-cell suspensions were passed through a 100-μm filter, and liver parenchyma cells were isolated in a 50% Percoll (Cytiva) density gradient, centrifuged at 70*g* for 10 min.

### Liver load assessment by RT–qPCR

C57BL/6 and BALB/c mice were killed, and the livers were collected at the indicated timepoints following infection/challenge, homogenized in PureZOL RNA Isolation Reagent (Bio-Rad) using the Mini-Beadbeater-16 cell disrupter (BioSpect) for 3 min, and stored at −80 °C until RNA extraction. Total RNA was extracted using the NZY Total RNA Isolation kit (NZYtech) according to the manufacturer’s instructions and quantified in a Nanodrop 1000 (Thermo Scientific). *Pb18s* and *Mus musculus Hprt* levels were assessed using TaqMan MGB probes (*Pb18s*: 5′ primer: AGGGAGCCTGAGAAATAG; 3′ primer: GTCACTACCTCTCTTATTTAGAA; probe: 6-FAM-ACCACATCTAAGGAAGGCAGCA-BHQ1; m*Hprt*: 5′ primer: GAACCAGGTTATGACCTA; 3′ primer: TCTCCTTCATGACATCTC; probe: 6-FAM-TTCAGTCCTGTCCATAATCAGTCCAT-BHQ1). One-step NZYSpeedy reverse transcription–qPCR (RT–qPCR) Probe kit (NZYtech) was used for tandem cDNA synthesis and amplification from 100 ng of total RNA, in a RT–PCR 7500 Fast cycler (Applied Biosystems). The programme consisted of 1 cycle at 50 °C for 20 min, 1 cycle at 95 °C for 5 min, followed by 40 cycles of 95 °C for 5 s and 60 °C for 1 min. The parameter threshold cycle (*C*t) was used to quantify the relative expression of both parasite and host genes with the comparative ΔΔ*C*t method, using *Mus musculus Hprt* as the normalizing reference gene.

### In vivo anti-CD8 antibody administration

RAS-immunized C57BL/6 and BALB/c mice received 200 mg of depleting anti-CD8 mouse monoclonal antibody (2.43; BioXCell) or IgG2b (LTF-2; BioXCell) diluted in saline via intraperitoneal route, 24 days after priming, and 4 days before the administration of 3 × 10^4^ infectious *Pb*WT SPZ.

### Isolation of splenocytes and hepatic leukocytes

C57BL/6 and BALB/c mice were killed at indicated timepoints, transcardially perfused with 15 ml of PBS, and the livers and spleens were collected and homogenized in PBS. Hepatic leukocytes were isolated in a 35% Percoll (Cytiva) density gradient, centrifuged at 1,360*g* for 20 min at room temperature. Subsequently, erythrocytes were lysed, and the total number of splenocytes and liver leukocytes was assessed in a 5-laser Cytek Aurora (Cytek Biosciences). Ex vivo cytokine production of CD8 T_RM_ cells was assessed following culture with phorbol 12-myristate 13-acetate (50 ng ml^−1^; Sigma) and ionomycin (500 ng ml^−1^; Sigma) for 4 h at 37 °C and 5% CO_2_, in the presence of brefeldin A (10 μg ml^−1^; Sigma).

### Flow cytometry

For high-parameter spectral flow cytometry analysis, hepatic leukocytes and splenocytes isolated from immunized cohorts were labelled with monoclonal antibodies specific for CD45 (30-F11; Biolegend), CD3 (17A2; Biolegend), NK1.1 (PK136; Biolegend), TCRδ (GL-3,GL3; eBiosciences), TCRβ (H57-597; Biolegend), CD11b (M1/70; Biolegend), CD4 (RM4-5; BD Biosciences), CD8 (53-6.7; BD Biosciences), CD62L (MEL-14; Biolegend), CD69 (H1.2F3; Biolegend), CD44 (IM7; Biolegend), KLRG1 (2F1; eBiosciences), CXCR6 (SA051D1; Biolegend), CXCR3 (CXCR3-173; Biolegend), CX3CR1 (SA011F11; Biolegend), CD127 (A7R34; Biolegend), PD-1 (29 F.1A12; Biolegend), CD11a (M17/4; BD Biosciences), CD11c (N418; Cytek Biosciences), MHC II (M5/114.15.2; BD Biosciences), F4/80 (BM8; Biolegend) and Ly6C (HK1.4; Cytek Biosciences). *Plasmodium*-specific CD8 T cells were immunophenotyped following labelling with H2-K^b^-*Pb*RPL6_120–127_ (NVFDFNNL; GenScript) and H2-D^b^-*Pb*TRAP_130–138_ (SALLNVDNL; GenScript) tetramers (NIH Tetramer Core Facility). Before surface staining, Fc receptors were blocked using a monoclonal antibody specific for CD16/CD32 (93; eBioscience), followed by incubation with H2-K^b^-*Pb*RPL6_120–127_ and H2-D^b^-*Pb*TRAP_130–138_ tetramers diluted in staining buffer (PBS with 2% FBS) for 60 min at 4 °C. Next, all antibodies were diluted in staining buffer, and staining was performed at 4 °C. Dead cells were excluded using a live–dead fixable dye (Cytek Biosciences or eBioscience), diluted in PBS and performed at 4 °C before incubation with surface or intracellular antibodies. Intracellular staining to evaluate cytokine production was performed using monoclonal antibodies specific for IFNγ (XMG1.2; Biolegend) and GzmB (REA226; Miltenyi Biotec) after cell fixation and permeabilization using the FoxP3/Transcription Factor Staining Kit (Invitrogen), according to the manufacturer’s instructions. Samples were acquired in a 5-laser Cytek Aurora (Cytek Biosciences), and unmixing and compensation corrections were performed using single-colour cell controls in SpectroFlo software version 3.3.0 (Cytek Biosciences). Data analysis was performed in FlowJo version 10.10.0 software (Tree Star).

Progression of blood-stage infection was evaluated by the daily assessment of parasitaemia, that is, the frequency of infected erythrocytes. For this, blood samples were collected from the tail vein in PBS and incubated for 10 min at 37 °C with monoclonal antibodies specific for Ter-119 (TER-119; eBioscience) and CD45 (30-F11; Biolegend), and Hoechst 33342 (Invitrogen), all diluted in PBS. The samples were acquired on a BD LSRFortessa (BD Biosciences), and compensation was performed using single-colour cell controls in FACSDiva software version 6.2.0 (BD Biosciences). Data analysis was performed in FlowJo verison 10.10.0 software (Tree Star). Frequency of infected erythrocytes was determined by the percentage of Ter-119^+^ CD45^−^ Hoechst^+^ single cells.

To evaluate CD8 T cell depletion, circulating leukocytes, hepatic leukocytes and splenocytes were isolated at the indicated timepoints, and stained with monoclonal antibodies specific for CD45 (30-F11; Biolegend), CD3 (17A2; Biolegend), CD4 (GK1.5; Biolegend) and CD8 (53-6.7; Biolegend). Fc receptors were blocked using a monoclonal antibody specific for CD16/CD32 (93; eBioscience). All antibodies were diluted in PBS. The samples were acquired on a BD LSRFortessa (BD Biosciences), and compensation was performed using single-colour cell controls in FACSDiva software version 6.2.0 (BD Biosciences). Data analysis was performed in FlowJo version 10.10.0 software (Tree Star).

To evaluate in vivo cell wounding, liver parenchyma cells were stained with a monoclonal antibody specific for CD31 (MEC13.3; Biolegend) and LIVE/DEAD Fixable Violet Dead Cell stain kit (Invitrogen). Cells were acquired in a BD LSRFortessa (BD Biosciences), and compensation was performed using single-colour cell controls in FACSDiva software version 6.2.0 (BD Biosciences). Data analysis was performed in FlowJo version 10.10.0 software (Tree Star).

### Hierarchical unsupervised analysis of spectral flow cytometry data

Analysis was performed using FlowJo verison 10.10.0 software (Tree Star). Abnormal events were excluded from all samples using the Peak Extraction and Cleaning Oriented Quality Control (PeacoQC) plugin. For the analysis of lymphocyte populations, a total of 80.000 live CD45^+^ CD3^+^ cells were randomly selected per condition, with equal numbers sampled from each mouse to ensure balanced representation. All cells (240,000 total) were concatenated into a single file. Dimensional reduction was performed using the UMAP plugin, followed by unsupervised FlowSOM hierarchical clustering based on the expression of CD8, CD4, NK1.1, TCRδ, CD62L, CD44, CD69, KLRG1 and CD11a. For the analysis of myeloid and NK cell populations, a total of 60,000 live CD45^+^ CD3^−^ cells expressing CD11b and/or NK1.1 were randomly selected from each condition, with equal numbers sampled from each mouse to ensure balanced representation. All cells (180,000 total) were concatenated into a single file. Dimensional reduction was again performed using the UMAP plugin. Hallmark markers used for hierarchical clustering with FlowSOM were MHCII, NK1.1, CD11b, F4/80, Ly6C and CD11c. For both concatenated files, several numbers of clusters were tested. To avoid over-clustering, phenotypically similar clusters were manually merged. Ultimately, 14 and 7 were selected, respectively, as best representing inter-cluster differences in marker expression.

### ELISA

Titres of circulating anti-SPZ IgG antibodies were assessed in serum at the indicated timepoints, as previously described^60^. In brief, serum was obtained by collecting 10 µl of blood from the tail vein of mice into 90 µl of PBS supplemented with heparin (10 U ml^−1^; Braun), followed by centrifugation for 2 min at 800*g*, at room temperature. Supernatants were stored at −20 °C until analysis. Dissected *Pb*WT SPZ were purified by nycodenz (Alere Technologies) gradient, and SPZ protein extracts were obtained by incubating 5 × 10^5^ purified SPZ in extraction buffer (150 mM NaCl, 20 mM Tris–HCl, 1% triton, 1 mM EDTA, pH of 7.5) and used to coat each 96-well Nunc Maxisorp ELISA plate (Sigma) overnight. Coated wells were incubated with blocking buffer (5% non-fat dry milk in PBS supplemented with 0.05% Tween 20 (Sigma-Aldrich) (PBST)) for 15 min on ice, after which 100 µl of diluted sera (1:1,000) was added to each well and incubated for 2 h at room temperature with continuous shaking. After washing with PBST, the samples were incubated with horeseradish peroxidase-conjugated goat anti-mouse IgG antibody (0.4 μg ml^−1^; Enzo Life Sciences) for 1 h at room temperature with continuous shaking. The plates were washed with PBST and incubated with TMB Substrate Reagent Set (BD OptEIA, BD Biosciences), according to the manufacturer’s instructions. The colourimetric reaction was stopped after 10 min by adding 50 µl of 2 M H_2_SO_4_, and absorbance was measured at an optical density of 450 nm using 570 nm as a reference wavelength in a Tecan Spark plate reader (Tecan).

### Statistical analysis

Statistically significant differences between two different groups were determined using the log-rank (Mantel–Cox) test, non-parametric two-tailed Mann–Whitney *t*-test, two-way repeated-measures ANOVA or the two-sided chi-squared test, as indicated. Where Mann–Whitney tests were used in panels containing three or more groups, comparisons were prespecified and hypothesis-driven, reflecting comparisons against a defined reference condition, and no correction for multiple comparisons was applied. All tests were carried out in GraphPad Prism software (version 8.4.3.; GraphPad).

### Material availability

This study generated a new *Pb* line genetically deficient for the *perforin-like protein 1* gene, which is available upon request.

### Reporting summary

Further information on research design is available in the Nature Portfolio Reporting Summary linked to this article.

## Supplementary information

Supplementary Information Supplementary_information_for_Extended_Data_Fig_1b.

Reporting Summary

## Source data

Source Data Fig. 1 Statistical source data and statistical analysis.

Source Data Fig. 2 Statistical source data and statistical analysis.

Source Data Fig. 3 Statistical source data and statistical analysis.

Source Data Fig. 4 Statistical source data and statistical analysis.

Source Data Extended Data Fig. 1 and Table 1 Statistical source data and statistical analysis.

Source Data Extended Data Fig. 2 and Table 2 Statistical source data and statistical analysis.

Source Data Extended Data Fig. 3 and Table 3 Statistical source data and statistical analysis.

Source Data Extended Data Fig. 4 and Table 4 Statistical source data and statistical analysis.

Source Data Extended Data Fig. 5 and Table 5 Statistical source data and statistical analysis.

## Figures and Tables

**Extended Data Fig. 6:**
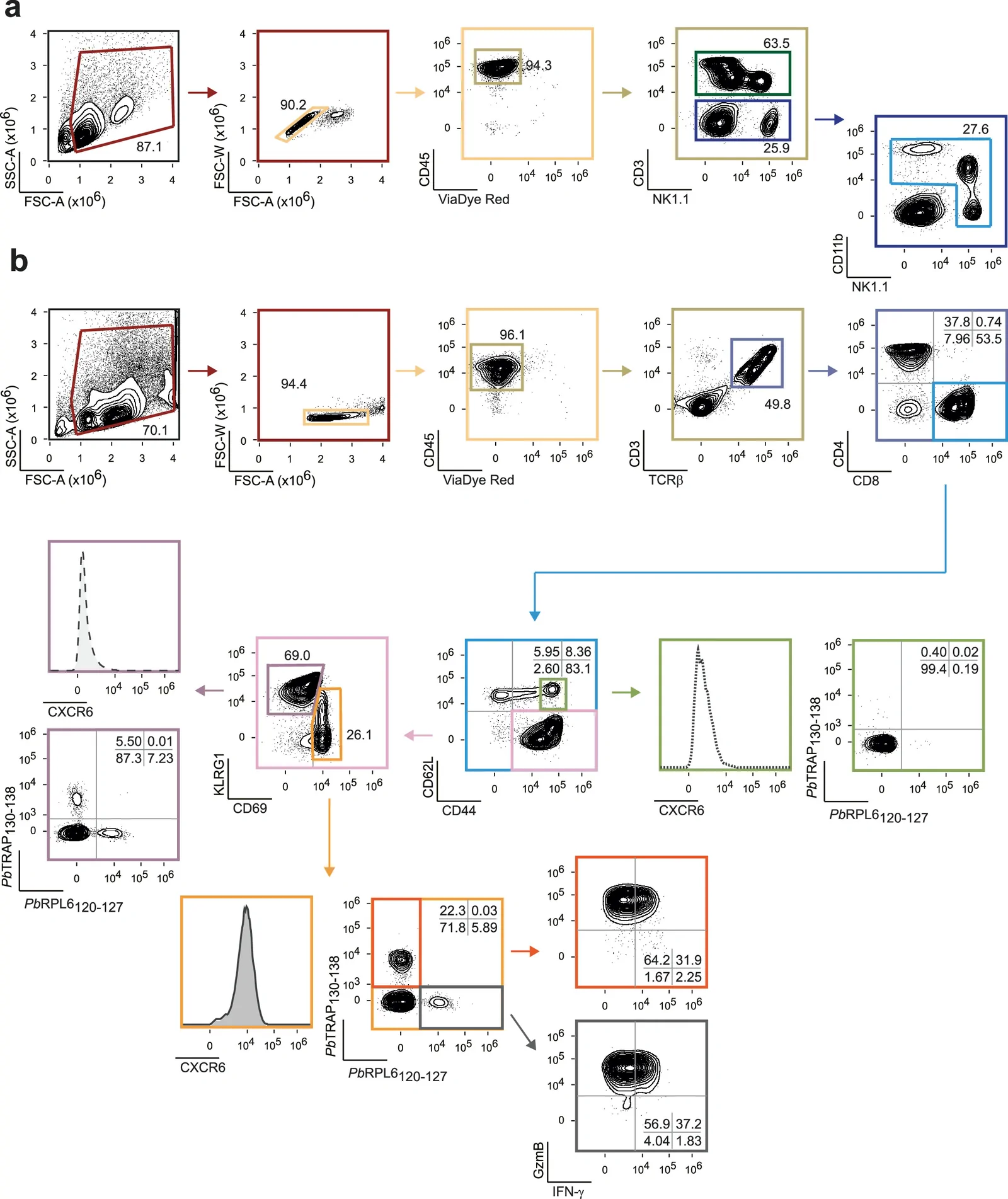
Gating strategies for main figures. **a**, Gating strategy used for downstream cell clustering of liver-isolated leukocytes. Leukocytes were gated using the forward scatter (FSC-A) and side scatter (SSC-A) parameters, cell aggregates were excluded using FSC-A *vs*. FSC-W, and live leukocytes were selected based on live/dead discrimination in a CD45 *vs*. ViaDye Red plot. Lymphocytes and natural killer T cells were selected for concatenation based on the expression of CD3 and NK1.1 in a CD3 *vs*. NK1.1 plot. CD3^−^ leukocytes were further gated based on the expression of CD11b and/or NK1.1 for concatenation and analysis of myeloid and natural killer subsets. **b**, Gating strategy used to evaluate *Plasmodium*-specific effector and memory CD8 T cell subsets. Leukocytes isolated from livers were gated using the forward scatter (FSC-A) and side scatter (SSC-A) parameters, cell aggregates were excluded using FSC-A *vs*. FSC-W, and live leukocytes were selected based on live/dead discrimination in a CD45 *vs*. ViaDye Red plot. Endogenous CD8 lymphocytes were selected based on the concomitant expression of CD3 and TCRβ and further gated in a CD8 *vs*. CD4 plot. CD8 lymphocytes were discriminated in a CD44 *vs*. CD62L plot in central memory (CD8 T_cm_) and activated cell subsets, with the later further specified in effector (CD8 T_eff_) and resident memory (CD8 T_rm_) cells in a KLRG1 *vs*. CD69 plot and expression of tissue residency markers (for example, CXCR6). Circulating and tissue resident *Plasmodium*-specific CD8 T cells were evaluated in a *Pb*TRAP_130-138_ *vs*. *Pb*RPL6_120-127_ plot, and their cytotoxic potential analyzed by the production of granzyme B *vs*. the production of IFN-γ.

**Extended Data Fig. 7:**
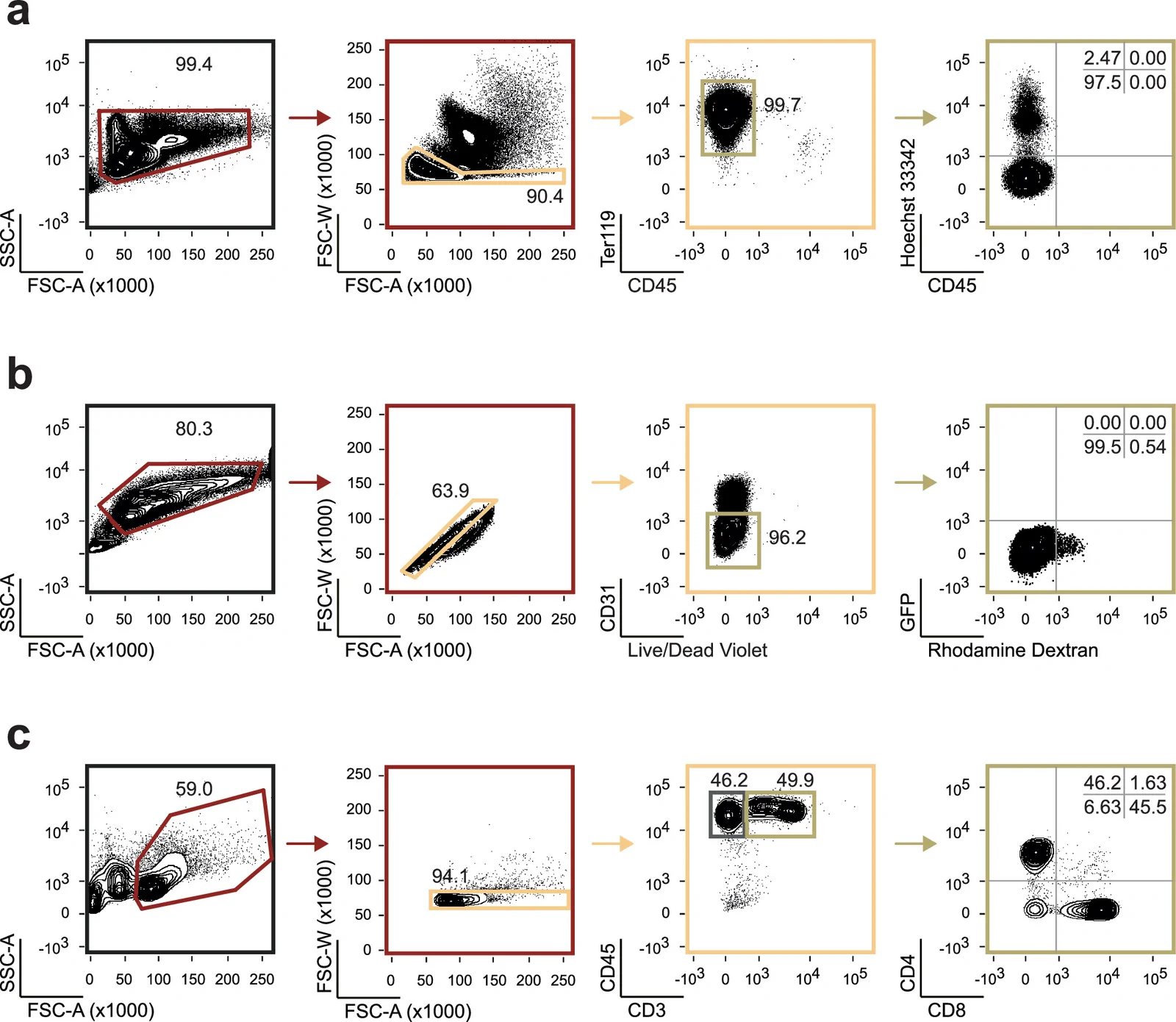
Gating strategies for Extended Data figures. **a**, Gating strategy used to analyze the frequency of infected erythrocytes (parasitemia). Erythrocytes were gated using the forward scatter (FSC-A) and side scatter (SSC-A) parameters, cell aggregates were excluded using FSC-A vs. FSC-W, and erythrocytes were selected based on Ter119 expression in a CD45 *vs*. TER119 plot. DNA content using Hoechst 33342 discriminated between infected and non-infected erythrocytes in a Hoechst 33342 *vs*. CD45 plot. **b**, Gating strategy used to analyze the frequency of traversed liver parenchyma cells. Liver parenchyma cells were gated using the forward scatter (FSC-A) and side scatter (SSC-A) parameters, cell aggregates were excluded using FSC-A *vs*. FSC-W, endothelial cells were excluded based on CD31 expression, and live parenchyma cells were selected in a CD31 *vs*. live/dead dye plot. Traversed cells were identified based on their content of rhodamine dextran in a GFP *vs*. rhodamine dextran plot. **c**, Gating strategy used to evaluate the depletion of CD8 T cells. Circulating leukocytes, or isolated from livers or spleens, were gated using the forward scatter (FSC-A) and side scatter (SSC-A) parameters, cell aggregates were excluded using FSC-A *vs*. FSC-W, and lymphocytes were selected based on CD45 and CD3 expression. Lymphocytes were further gated according to the expression of CD8 and CD4.

## Data Availability

All data are available in the Article and in the Source data, and can be assessed at https://doi.org/10.17632/cgfmnssg59.1Source data are provided with this paper.

## References

[1] World Health Organization. *World Malaria Report 2025* (2025).

[2] Langhorne, J., Ndungu, F. M., Sponaas, A. M. & Marsh, K. Immunity to malaria: more questions than answers. *Nat. Immunol.* **9**, 725–732 (2008). 10.1038/ni.f.205 18563083

[3] Duffy, P. E. & Patrick Gorres, J. Malaria vaccines since 2000: progress, priorities, products. *npj Vaccines* **5**, 48 (2020). 10.1038/s41541-020-0196-3 PMC7283239 32566259

[4] Mota, M. M. et al. Migration of *Plasmodium* Sporozoites through cells before infection. *Science* **20**, 141–144 (2001). 10.1126/science.291.5501.141 11141568

[5] Risco-Castillo, V. et al. Malaria sporozoites traverse host cells within transient vacuoles. *Cell Host Microbe* **18**, 593–603 (2015). 10.1016/j.chom.2015.10.006 26607162

[6] The RTS, S. C. T. P. A Phase 3 trial of RTS,S/AS01 malaria vaccine in African infants. *N. Engl. J. Med.* **367**, 2284–2295 (2012). 10.1056/NEJMoa1208394 PMC10915853 23136909

[7] Datoo, M. S. et al. Efficacy of a low-dose candidate malaria vaccine, R21 in adjuvant Matrix-M, with seasonal administration to children in Burkina Faso: a randomised controlled trial. *Lancet* **397**, 1809–1818 (2021). 10.1016/S0140-6736(21)00943-0 PMC8121760 33964223

[8] Datoo, M. S. et al. Safety and efficacy of malaria vaccine candidate R21/Matrix-M in African children: a multicentre, double-blind, randomised, phase 3 trial. *Lancet* **403**, 533–544 (2024). 10.1016/S0140-6736(23)02511-4 PMC7618965 38310910

[9] The RTS, S. C. T. P. Efficacy and safety of RTS,S/AS01 malaria vaccine with or without a booster dose in infants and children in Africa: final results of a phase 3, individually randomised, controlled trial. *Lancet* **386**, 31–45 (2015). 10.1016/S0140-6736(15)60721-8 PMC5626001 25913272

[10] Sissoko, M. S. et al. Safety and efficacy of a three-dose regimen of Plasmodium falciparum sporozoite vaccine in adults during an intense malaria transmission season in Mali: a randomised, controlled phase 1 trial. *Lancet Infect. Dis.* **22**, 377–389 (2022). 10.1016/S1473-3099(21)00332-7 PMC8981424 34801112

[11] Mwakingwe-Omari, A. et al. Two chemoattenuated PfSPZ malaria vaccines induce sterile hepatic immunity. *Nature* **595**, 289–294 (2021). 10.1038/s41586-021-03684-z PMC11127244 34194041

[12] Moita, D. et al. The effect of dosage on the protective efficacy of whole-sporozoite formulations for immunization against malaria. *npj Vaccines* **8**, 182 (2023). 10.1038/s41541-023-00778-9 PMC10667361 37996533

[13] Kreutzfeld, O., Müller, K. & Matuschewski, K. Engineering of genetically arrested parasites (GAPs) for a precision malaria vaccine. *Front. Cell. Infect. Microbiol.* **7**, 198 (2017). 10.3389/fcimb.2017.00198 PMC5450620 28620583

[14] Goswami, D., Minkah, N. K. & Kappe, S. H. I. Designer parasites: genetically engineered *plasmodium* as vaccines to prevent malaria infection. *J. Immunol.* **202**, 20–28 (2019). 10.4049/jimmunol.1800727 30587570

[15] Mo, A. X., McGugan, G. & Pesce, J. T. Meeting report: expert consultation on late arresting replication competent (LARC) malaria sporozoite vaccine research & development. *Vaccine* **54**, 127009 (2025). 10.1016/j.vaccine.2025.127009 40245769

[16] Seder, R. A. et al. Protection against malaria by intravenous immunization with a nonreplicating sporozoite vaccine. *Science* **341**, 1359–1365 (2013). 10.1126/science.1241800 23929949

[17] Roestenberg, M. et al. A double-blind, placebo-controlled phase 1/2a trial of the genetically attenuated malaria vaccine PfSPZ-GA1. *Sci. Transl. Med.* **12**, 544 (2020). 10.1126/scitranslmed.aaz5629 32434847

[18] Ishino, T., Yano, K., Chinzei, Y. & Yuda, M. Cell-passage activity is required for the malarial parasite to cross the liver sinusoidal cell layer. *PLoS Biol.* **2**, E4 (2004). 10.1371/journal.pbio.0020004 PMC314464 14737184

[19] Ishino, T., Chinzei, Y. & Yuda, M. A Plasmodium sporozoite protein with a membrane attack complex domain is required for breaching the liver sinusoidal cell layer prior to hepatocyte infection. *Cell. Microbiol.* **7**, 199–208 (2005). 10.1111/j.1462-5822.2004.00447.x 15659064

[20] Nganou-Makamdop, K., van Gemert, G.-J., Arens, T., Hermsen, C. C. & Sauerwein, R. W. Long term protection after immunization with *P. berghei* sporozoites correlates with sustained IFNγ responses of hepatic CD8+ memory T cells. *PLoS ONE* **7**, e36508 (2012). 10.1371/journal.pone.0036508 PMC3341355 22563506

[21] Butler, N. S., Vaughan, A. M., Harty, J. T. & Kappe, S. H. I. Whole parasite vaccination approaches for prevention of malaria infection. *Trends Immunol.* **33**, 247–254 (2012). 10.1016/j.it.2012.02.001 22405559

[22] Matuschewski, K. Vaccines against malaria—still a long way to go. *FEBS J.* **284**, 2560–2568 (2017). 10.1111/febs.14107 28500775

[23] Friesen, J. & Matuschewski, K. Comparative efficacy of pre-erythrocytic whole organism vaccine strategies against the malaria parasite. *Vaccine* **29**, 7002–7008 (2011). 10.1016/j.vaccine.2011.07.034 21787828

[24] Moita, D. & Prudêncio, M. Whole-sporozoite malaria vaccines: where we are, where we are going. *EMBO Mol. Med.* **16**, 2279–2289 (2024). 10.1038/s44321-024-00131-0 PMC11473726 39284948

[25] Ishino, T., Chinzei, Y. & Yuda, M. Two proteins with 6-cys motifs are required for malarial parasites to commit to infection of the hepatocyte. *Mol. Microbiol.* **58**, 1264–1275 (2005). 10.1111/j.1365-2958.2005.04801.x 16313615

[26] Abuga, K. M., Jones-Warner, W. & Hafalla, J. C. R. Immune responses to malaria pre-erythrocytic stages: implications for vaccine development. *Parasite Immunol.* **43**, e12795 (2021). 10.1111/pim.12795 PMC7612353 32981095

[27] Aguirre-Botero, M. C. et al. Late killing of *Plasmodium berghei* sporozoites in the liver by an anti-circumsporozoite protein antibody. *eLife* **14**, RP105291 (2025). 10.7554/eLife.105291 PMC11828480 39951341

[28] Keitany, G. J. et al. Immunization of mice with live-attenuated late liver stage-arresting plasmodium yoelii parasites generates protective antibody responses to preerythrocytic stages of malaria. *Infect. Immun.* **82**, 5143–5153 (2014). 10.1128/IAI.02320-14 PMC4249261 25267837

[29] Guyach, S. E., Bryan, M. A. & Norris, K. A. Differences in antibody and immune responses between Balb/c and C57Bl/6 mice infected with *Trypanosoma cruzi* (129.5). *J. Immunol.* **182**, 129.5–129.5 (2009).

[30] Zeng, M., Nourishirazi, E., Guinet, E. & Nouri-Shirazi, M. The genetic background influences the cellular and humoral immune responses to vaccines. *Clin. Exp. Immunol.* **186**, 190–204 (2016). 10.1111/cei.12841 PMC5054571 27393001

[31] Chu, Y. et al. CpG adjuvant enhances humoral and cellular immunity against OVA in different degrees in BALB/c, C57BL/6J, and C57BL/6N mice. *Int. Immunopharmacol.* **138**, 112593 (2024). 10.1016/j.intimp.2024.112593 38972210

[32] Fernandez-Ruiz, D. et al. Liver-resident memory CD8^+^ T cells form a front-line defense against malaria liver-stage infection. *Immunity* **45**, 889–902 (2016). 10.1016/j.immuni.2016.08.011 27692609

[33] Holz, L. E. et al. CD8^+^ T cell activation leads to constitutive formation of liver tissue-resident memory t cells that seed a large and flexible niche in the liver. *Cell Rep.* **25**, 68–79.e4 (2018). 10.1016/j.celrep.2018.08.094 30282039

[34] Doolan, D. L. & Hoffman, S. L. IL-12 and NK cells are required for antigen-specific adaptive immunity against malaria initiated by CD8 + T cells in the *Plasmodium yoelii* model. *J. Immunol.* **163**, 884–892 (1999). 10395683

[35] Miller, J. L., Sack, B. K., Baldwin, M., Vaughan, A. M. & Kappe, S. H. I. Interferon-mediated innate immune responses against malaria parasite liver stages. *Cell Rep.* **7**, 436–447 (2014). 10.1016/j.celrep.2014.03.018 24703850

[36] Bartsch, L. M., Damasio, M. P. S., Subudhi, S. & Drescher, H. K. Tissue-resident memory T cells in the liver—unique characteristics of local specialists. *Cells* **9**, 2457 (2020). 10.3390/cells9112457 PMC7696520 33187162

[37] Devi, K. S. P. et al. PD-1 is requisite for skin T_RM_ cell formation and specification by TGF. *Nat. Immunol.* **26**, 1339–1351 (2025). 10.1038/s41590-025-02228-1 PMC12307224 40730902

[38] Gerlach, C. et al. The chemokine receptor CX3CR1 defines three antigen-experienced CD8 T cell subsets with distinct roles in immune surveillance and homeostasis. *Immunity* **45**, 1270–1284 (2016). 10.1016/j.immuni.2016.10.018 PMC5177508 27939671

[39] de Menezes, M. N. et al. Long lived liver-resident memory T cells of biased specificities for abundant sporozoite antigens drive malaria protection by radiation-attenuated sporozoite vaccination. *PLoS Pathog* **21**, e1012731 (2025). 10.1371/journal.ppat.1012731 PMC12143544 40424562

[40] Hafalla, J. C. R. et al. Identification of targets of CD8^+^ T cell responses to malaria liver stages by genome-wide epitope profiling. *PLoS Pathog.* **9**, e1003303 (2013). 10.1371/journal.ppat.1003303 PMC3649980 23675294

[41] Lau, L. S. et al. CD8^+^ T cells from a novel T cell receptor transgenic mouse induce liver-stage immunity that can be boosted by blood-stage infection in rodent malaria. *PLoS Pathog.* **10**, e1004135 (2014). 10.1371/journal.ppat.1004135 PMC4031232 24854165

[42] Amino, R. et al. Quantitative imaging of *Plasmodium* transmission from mosquito to mammal. *Nat. Med.* **12**, 220–224 (2006). 10.1038/nm1350 16429144

[43] Ejigiri, I. et al. Shedding of TRAP by a rhomboid protease from the malaria sporozoite surface is essential for gliding motility and sporozoite infectivity. *PLoS Pathog.* **8**, e1002725 (2012). 10.1371/journal.ppat.1002725 PMC3406075 22911675

[44] Valencia-Hernandez, A. M. et al. A natural peptide antigen within the *Plasmodium* ribosomal protein RPL6 confers liver TRM cell-mediated immunity against malaria in mice. *Cell Host Microbe* **27**, 950–962.e7 (2020). 10.1016/j.chom.2020.04.010 32396839

[45] Howick, V. M. et al. The Malaria Cell Atlas: single parasite transcriptomes across the complete *Plasmodium* life cycle. *Science* **365**, eaaw2619 (2019). 10.1126/science.aaw2619 PMC7056351 31439762

[46] Hall, N. et al. A comprehensive survey of the *Plasmodium* life cycle by genomic, transcriptomic, and proteomic analyses. *Science* **307**, 82–86 (2005). 10.1126/science.1103717 15637271

[47] Vanderberg, J., Nussenzweig, R. & Most, H. Protective immunity produced by the injection of x-irradiated sporozoites of *Plasmodium berghei*. V. In vitro effects of immune serum on sporozoites. *Mil. Med.* **134**, 1183–1190 (1969). 4987037

[48] Sano, G. et al. Swift development of protective effector functions in naive Cd8+ T cells against malaria liver stages. *J. Exp. Med.* **194**, 173–180 (2001). 10.1084/jem.194.2.173 PMC2193458 11457892

[49] Pohl, K. G. et al. Innate immune sensing of cell traversal by *Plasmodium* sporozoites drives protective T cell responses. *Nat. Micro.* 10.1038/s41564-026-02460-x (2026). 42816580

[50] Tavares, J. et al. Role of host cell traversal by the malaria sporozoite during liver infection. *J. Exp. Med.* **210**, 905–915 (2013). 10.1084/jem.20121130 PMC3646492 23610126

[51] Formaglio, P., Tavares, J., Ménard, R. & Amino, R. Loss of host cell plasma membrane integrity following cell traversal by *Plasmodium* sporozoites in the skin. *Parasitol. Int.* **63**, 237–244 (2014). 10.1016/j.parint.2013.07.009 23892177

[52] Kanatani, S., Stiffler, D., Bousema, T., Yenokyan, G. & Sinnis, P. Revisiting the *Plasmodium* sporozoite inoculum and elucidating the efficiency with which malaria parasites progress through the mosquito. *Nat. Commun.* **15**, 748 (2024). 10.1038/s41467-024-44962-4 PMC10811227 38272943

[53] Nunes-Cabaço, H., Moita, D. & Prudêncio, M. Five decades of clinical assessment of whole-sporozoite malaria vaccines. *Front. Immunol.* **13**, 977472 (2022). 10.3389/fimmu.2022.977472 PMC9493004 36159849

[54] Kazmin, D. et al. Systems analysis of protective immune responses to RTS,S malaria vaccination in humans. *Proc. Natl Acad. Sci. USA* **114**, 2425–2430 (2017). 10.1073/pnas.1621489114 PMC5338562 28193898

[55] Janse, C. J. et al. High efficiency transfection of *Plasmodium berghei* facilitates novel selection procedures. *Mol. Biochem. Parasitol.* **145**, 60–70 (2006). 10.1016/j.molbiopara.2005.09.007 16242190

[56] Billker, O. et al. Calcium and a calcium-dependent protein kinase regulate gamete formation and mosquito transmission in a malaria parasite. *Cell* **117**, 503–514 (2004). 10.1016/s0092-8674(04)00449-0 15137943

[57] Zuzarte-Luis, V., Sales-Dias, J. & Mota, M. M. Simple, sensitive and quantitative bioluminescence assay for determination of malaria pre-patent period. *Malar. J.* **13**, 15 (2014). 10.1186/1475-2875-13-15 PMC3893453 24400642

[58] Bento, I. et al. Parasite and vector circadian clocks mediate efficient malaria transmission. *Nat. Microbiol.* **10**, 882–896 (2025). 10.1038/s41564-025-01949-1 PMC11964930 40164831

[59] Afriat, A. et al. A spatiotemporally resolved single-cell atlas of the *Plasmodium* liver stage. *Nature* **611**, 563–569 (2022). 10.1038/s41586-022-05406-5 36352220

[60] Nunes-Cabaço, H., Moita, D., Rôla, C., Mendes, A. M. & Prudêncio, M. Impact of dietary protein restriction on the immunogenicity and efficacy of whole-sporozoite malaria vaccination. *Front. Immunol.* **13**, 869757 (2022). 10.3389/fimmu.2022.869757 PMC9070679 35529859

