# Supplementary material for: Host cell traversal by Plasmodium parasites drives sterilizing T cell-mediated immunity following immunization
Source: Nat Microbiol. 2026 Sep 30;11(11):3180–92. doi: 10.1038/s41564-026-02477-2 (PMC13739723; doi:10.1038/s41564-026-02477-2)
Supplement: Supplementary file 1 [file 41564_2026_2477_MOESM1_ESM.pdf]

nature microbiology

Supplementary information

https://doi.org/10.1038/s41564-026-02477-2

Host cell traversal by *Plasmodium* parasites drives sterilizing T cell-mediated immunity following immunization

In the format provided by the authors and unedited

Supplementary information for Extended Data Fig. 1b – Uncropped agarose gel pertaining to panel 1b.

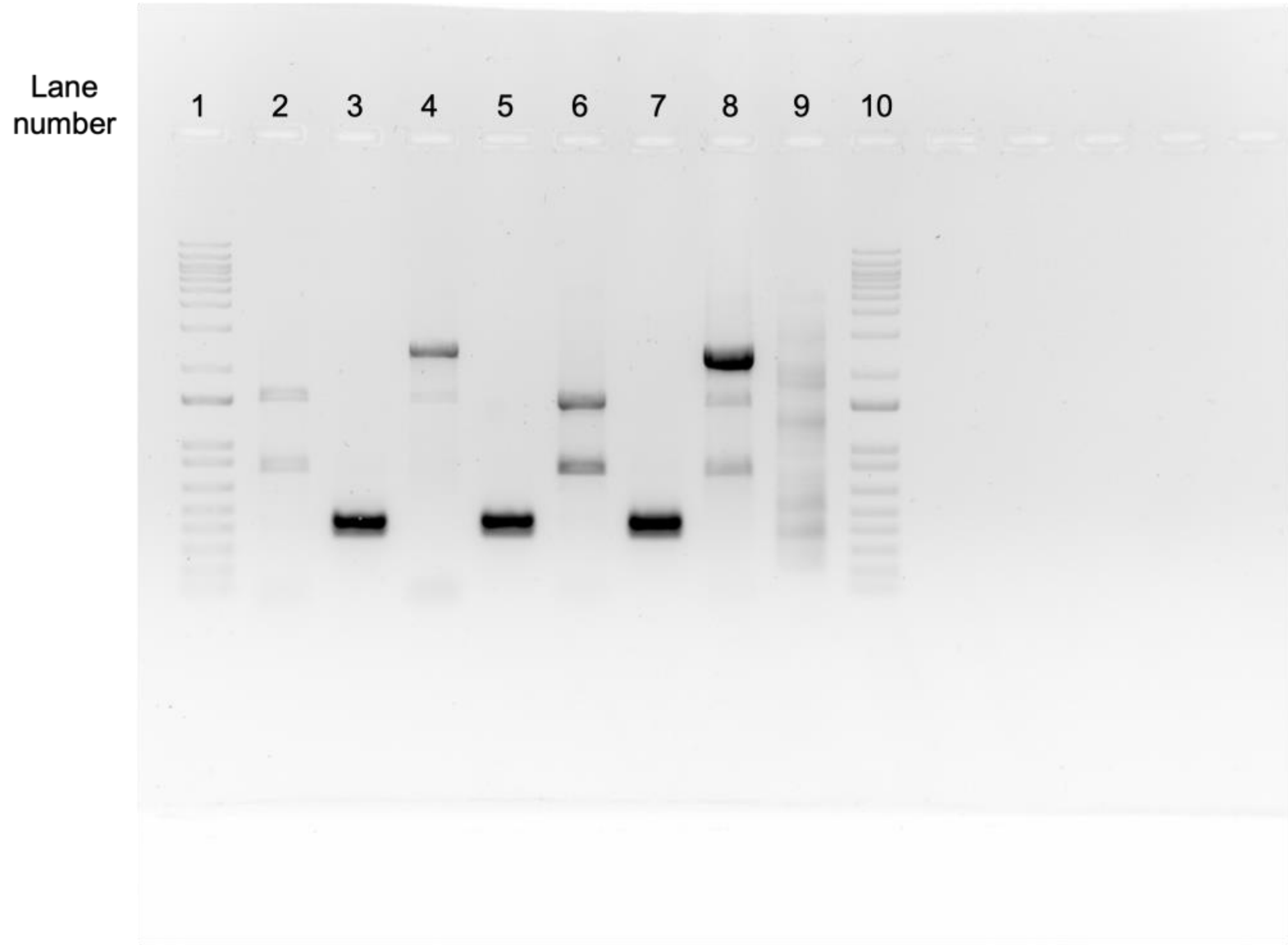

Legend:

Lane 1 and 10 – Molecular weight marker

Lane 2, 4, 6 and 8 - Cassette integration PCR;

Lane 3, 5, 7 and 9 - WT locus PCR
