# Supplementary material for: Host cell traversal by Plasmodium parasites drives sterilizing T cell-mediated immunity following immunization
Source: Nat Microbiol. 2026 Sep 30;11(11):3180–92. doi: 10.1038/s41564-026-02477-2 (PMC13739723; doi:10.1038/s41564-026-02477-2)
Supplement: Supplementary file 2 [file 41564_2026_2477_MOESM2_ESM.pdf]

nature portfolio

Corresponding author(s): Maria M. Mota and Ângelo Ferreira Chora

Last updated by author(s): Jul 24, 2026

Reporting Summary

Nature Portfolio wishes to improve the reproducibility of the work that we publish. This form provides structure for consistency and transparency in reporting. For further information on Nature Portfolio policies, see our Editorial Policies and the Editorial Policy Checklist.

Statistics

For all statistical analyses, confirm that the following items are present in the figure legend, table legend, main text, or Methods section.

n/a	Confirmed	
☐	☒	The exact sample size (*n*) for each experimental group/condition, given as a discrete number and unit of measurement
☐	☒	A statement on whether measurements were taken from distinct samples or whether the same sample was measured repeatedly
☐	☒	The statistical test(s) used AND whether they are one- or two-sided<br>*Only common tests should be described solely by name; describe more complex techniques in the Methods section.*
☒	☐	A description of all covariates tested
☒	☐	A description of any assumptions or corrections, such as tests of normality and adjustment for multiple comparisons
☐	☒	A full description of the statistical parameters including central tendency (e.g. means) or other basic estimates (e.g. regression coefficient) AND variation (e.g. standard deviation) or associated estimates of uncertainty (e.g. confidence intervals)
☐	☒	For null hypothesis testing, the test statistic (e.g. *F*, *t*, *r*) with confidence intervals, effect sizes, degrees of freedom and *P* value noted<br>*Give P values as exact values whenever suitable.*
☒	☐	For Bayesian analysis, information on the choice of priors and Markov chain Monte Carlo settings
☒	☐	For hierarchical and complex designs, identification of the appropriate level for tests and full reporting of outcomes
☒	☐	Estimates of effect sizes (e.g. Cohen's *d*, Pearson's *r*), indicating how they were calculated

Our web collection on statistics for biologists contains articles on many of the points above.

Software and code

Policy information about availability of computer code

Data collection	Flow Cytometry: Samples were acquired in a 5-laser Cytek Aurora (Cytek Biosciences) or a BD LSRFortessa (BD Biosciences) analyzer, using the SpectroFlo software V3.3.0 (Cytek Biosciences) or the FACSDiva software V6.2.0 (BD Biosciences), respectively.<br>In vivo Bioluminescence: In vivo functional luciferase activity was acquired using the IVIS Lumina imaging system (PerkinElmer).<br>Confocal Microscopy: Images were acquired in a Zeiss LSM 880 with Airyscan microscope (Zeiss) and a Zeiss LSM 980.32 with Airyscan microscope (Zeiss) using the ZEN 2.3 SP1 (black edition).
Data analysis	Flow Cytometry: Data analysis was performed in FlowJo V10.10.0 software (Tree Star Inc.). For the hierarchical unsupervised analysis, the Peak Extraction and Cleaning Oriented Quality Control (PeacoQC) plugin was used to exclude abnormal events. The UMAP plugin was then used to perform dimensional reduction, followed by unsupervised hierarchical clustering using the FlowSOM plugin.<br>In vivo Bioluminescence: Average radiance was quantified using the Living Image 3.0 software (PerkinElmer).<br>Confocal Microscopy: Images were analyzed using Fiji Image J (NIH).<br>Statistical analyses: Statistically significant differences were assessed out in GraphPad Prism software (V8.4.3.; GraphPad).

For manuscripts utilizing custom algorithms or software that are central to the research but not yet described in published literature, software must be made available to editors and reviewers. We strongly encourage code deposition in a community repository (e.g. GitHub). See the Nature Portfolio guidelines for submitting code & software for further information.

Data

Policy information about availability of data

All manuscripts must include a data availability statement. This statement should provide the following information, where applicable:

- Accession codes, unique identifiers, or web links for publicly available datasets
- A description of any restrictions on data availability
- For clinical datasets or third party data, please ensure that the statement adheres to our policy

All data are available in the main text and in the source data, and can be assessed at https://data.mendeley.com/preview/cgfmnssg59?a=09c74595-5246-4b89-b37c-7197bdb672a7

Research involving human participants, their data, or biological material

Policy information about studies with human participants or human data. See also policy information about sex, gender (identity/presentation), and sexual orientation and race, ethnicity and racism.

Reporting on sex and gender	*Use the terms sex (biological attribute) and gender (shaped by social and cultural circumstances) carefully in order to avoid confusing both terms. Indicate if findings apply to only one sex or gender; describe whether sex and gender were considered in study design; whether sex and/or gender was determined based on self-reporting or assigned and methods used. Provide in the source data disaggregated sex and gender data, where this information has been collected, and if consent has been obtained for sharing of individual-level data; provide overall numbers in this Reporting Summary. Please state if this information has not been collected. Report sex- and gender-based analyses where performed, justify reasons for lack of sex- and gender-based analysis.*
Reporting on race, ethnicity, or other socially relevant groupings	*Please specify the socially constructed or socially relevant categorization variable(s) used in your manuscript and explain why they were used. Please note that such variables should not be used as proxies for other socially constructed/relevant variables (for example, race or ethnicity should not be used as a proxy for socioeconomic status). Provide clear definitions of the relevant terms used, how they were provided (by the participants/respondents, the researchers, or third parties), and the method(s) used to classify people into the different categories (e.g. self-report, census or administrative data, social media data, etc.) Please provide details about how you controlled for confounding variables in your analyses.*
Population characteristics	*Describe the covariate-relevant population characteristics of the human research participants (e.g. age, genotypic information, past and current diagnosis and treatment categories). If you filled out the behavioural & social sciences study design questions and have nothing to add here, write "See above."*
Recruitment	*Describe how participants were recruited. Outline any potential self-selection bias or other biases that may be present and how these are likely to impact results.*
Ethics oversight	*Identify the organization(s) that approved the study protocol.*

Note that full information on the approval of the study protocol must also be provided in the manuscript.

Field-specific reporting

Please select the one below that is the best fit for your research. If you are not sure, read the appropriate sections before making your selection.

☒ Life sciences ☐ Behavioural & social sciences ☐ Ecological, evolutionary & environmental sciences

For a reference copy of the document with all sections, see nature.com/documents/nr-reporting-summary-flat.pdf

Life sciences study design

All studies must disclose on these points even when the disclosure is negative.

Sample size	Sample size on mice experiments were chosen on the basis of historical data, established by previous studies conducted by this laboratory.
Data exclusions	No experimental animals were excluded.
Replication	All attempts of replication were successful, the observed variation is reported and contributes to the statistical calculation. Biological replicates are enumerated in the manuscript.
Randomization	Experimental animals were randomly assigned to the different experimental groups.
Blinding	All data were collected and analyzed in a non-blind fashion due to the non-subjective nature of the measurements.

Reporting for specific materials, systems and methods

We require information from authors about some types of materials, experimental systems and methods used in many studies. Here, indicate whether each material, system or method listed is relevant to your study. If you are not sure if a list item applies to your research, read the appropriate section before selecting a response.

Materials & experimental systems

n/a	Involved in the study
☐	☒ Antibodies
☐	☒ Eukaryotic cell lines
☒	☐ Palaeontology and archaeology
☐	☒ Animals and other organisms
☒	☐ Clinical data
☒	☐ Dual use research of concern
☒	☐ Plants

Methods

n/a	Involved in the study
☒	☐ ChIP-seq
☐	☒ Flow cytometry
☒	☐ MRI-based neuroimaging

Antibodies

Antibodies used

Flow Cytometry:
Unconjugated anti-mouse CD16/CD32, clone 93 eBiosciences; 1:50
PerCP-Cy5.5 anti-mouse Ter119, clone TER-119 eBiosciences; 1:400
APC anti-mouse CD45, clone 30-F11 Biolegend; 1:400
Alexa Fluor 700 anti-mouse CD45, clone 30-F11 Biolegend; 1:300
BV785 anti-mouse CD45.2, clone 104 Biolegend; 1:200
BUV496 anti-mouse CD8, clone 53-6.7 BD Biosciences; 1:100
BV711 anti-mouse CD8, clone 53-6.7 Biolegend; 1:400
Biotin anti-mouse CD8, clone 53-6.7 Biolegend; 1:550
BUV563 anti-mouse CD11a, clone M17/4 BD Biosciences; 1:300
BV510 anti-mouse CD4, clone GK1.5 Biolegend; 1:400
BUV805 anti-mouse CD4, clone RM4-5 BD Biosciences; 1:100
BV421 anti-mouse IFN-g, clone XMG1.2 Biolegend; 1:100
cFluor V450 anti-mouse CD11c, clone N418 Cytek Biosciences; 1:100
BV480 anti-mouse I-A/I-E, clone M5/114.15.2 BD Biosciences; 1:100
BV510 anti-mouse CD62L, clone MEL-14 Biolegend; 1:300
BV605 anti-mouse/human CD44, clone IM7 Biolegend; 1:200
BV785 anti-mouse/human CD44, clone IM7 Biolegend; 1:300
BV650 anti-mouse CD69, clone H1.2F3 Biolegend; 1:100
BV711 anti-mouse NK-1.1, clone PK136 Biolegend; 1:100
PE anti-mouse NK1.1, clone PK136 CyFlow; 1:500
Vio B515 anti-human/rat/mouse Granzyme B, clone REA226 Miltenyi Biotec; 1:100
PerCP-eFluor 710 anti-mouse KLRG1, clone 2F1 eBiosciences; 1:200
FITC anti-mouse/human CD11b, clone M1/70 Biolegend; 1:400
PE/Dazzle 594 anti-mouse F4/80, clone BM8 Biolegend; 1:200
PE-Cy5 anti-mouse TCR gamma/delta, clone eBioGL3(GL-3, GL3) eBiosciences; 1:200
BV421 anti-mouse TCR gamma/delta, clone GL3 Biolegend; 1:100
PE-Cy7 TCR beta, clone H57-597 Biolegend; 1:100
PE-Cy5 TCR beta, clone H57-597 Biolegend; 1:200
PE-Cy7 anti-mouse CD3, clone 17A2 Biolegend; 1:100
PE anti-mouse CD3, clone 17A2 Biolegend; 1:200
BV711 anti-mouse CXCR6, clone SA051D1 Biolegend; 1:100
APC anti-mouse CXCR3, clone CXCR3-173 Biolegend; 1:100
Alexa Fluor 647 anti-mouse CX3CR1, clone Z8-50.23 eBiosciences; 1:200
PE-Cy5 anti-mouse PD-1, clone 29F.1A12 Biolegend; 1:300
FITC anti-mouse CD127, A7R34 Biolegend; 1:200
APC anti-mouse CD31, clone MEC13.3 Biolegend; 1:400
cFluor R840 anti-mouse Ly6C, clone HK1.4 Cytek Biosciences; 1:100
BV421 anti-PbTRAP 130-138, SALLNVDNL Genscript, NIH tetramer facility; 1:100
APC anti-PbRPL6 120-127, NVFDFNNL Genscript, NIH tetramer facility; 1:100

In vivo depletions:
InVivo MAb anti-mouse CD8, clone 2.43 BioXCell; 200ug per mouse
InVivo MAb rat IgG2b isotype control, anti-keyhole limpet hemocyanin, clone LTF-2 BioXCell; 200ug per mouse

Confocal Microscopy:
Alexa Fluor 488 rabbit polyclonal antibody anti-GFP, Invitrogen; 1:500
Unconjugated polyclonal antibody goat anti-UIS4, Sicgen; 1:500
Alexa Fluor 568 donkey anti-goat IgG (H+L) polyclonal antibody, Invitrogen; 1:500
Alexa Fluor 568 donkey anti-rabbit IgG (H+L) polyclonal antibody, Invitrogen; 1:500

	Alexa Fluor 647 donkey anti-mouse IgG (H+L) polyclonal antibody, Invitrogen; 1:500<br><br>ELISA:<br>HRP conjugated, goat anti-mouse IgG F(ab')2, polyclonal antibody, Enzo Life Sciences; 1:2000
Validation	Flow Cytometry: Fluorochrome-conjugated monoclonal antibodies were purchased from commercial vendors. Any information regarding validation of conjugated antibodies for use in flow cytometry is retrievable from the vendors' website.<br><br>In vivo depletions: BIO X CELL warrants that the InVivo MAb anti-mouse CD8 depleting antibody binds to its target antigen, as stated in the Technical Data Sheet found at found at https://bioxcell.com/pub/media/tds/BE0061-TDS.pdf. In addition, we also validated efficient CD8 T cell depletion following InVivo MAb anti-mouse CD8 antibody by flow cytometry.<br><br>Confocal Microscopy: The laboratory has previously validated the use of Alexa Fluor 488 anti-GFP, unconjugated goat anti-UIS4 and Alexa Fluor 568 donkey anti-goat IgG antibodies in https://doi.org/10.1038/s41564-025-01949-1 and https://doi.org/10.1016/j.celrep.2022.110886

Eukaryotic cell lines

Policy information about cell lines and Sex and Gender in Research

Cell line source(s)	Parasites:<br>Plasmodium berghei ANKA GFP-LucCON (676m1cl1; RMgm-29) DOI:10.1016/j.molbiopara.2005.09.007; Propagated in house<br>Plasmodium berghei ANKA GFPCON (2.34) DOI: 10.1016/s0092-8674(04)00449-0; Propagated in house<br>Plasmodium berghei ANKA Spect1- (RMgm-138) DOI: 10.1371/journal.pbio.0020004; Propagated in house<br>Plasmodium berghei ANKA Plp1- this paper; Propagated in house<br>Plasmodium berghei ANKA P36p- (417m0cl2; RMgm-41) DOI:10.1111/j.1365-2958.2005.04801.x; Propagated in house<br><br>Mammalian cells:<br>HepG2 cells were purchased from ATCC.
Authentication	Parasite fitness was confirmed by functional and phenotypical characterization during infections both in vitro and in vivo by evaluating infectivity, cell traversal motility, intrahepatic development and malaria establishment (in vivo).
Mycoplasma contamination	All cell lines were monitored for Mycoplasma contamination, and found to be negative.
Commonly misidentified lines<br>(See ICLAC register)	None

Animals and other research organisms

Policy information about studies involving animals; ARRIVE guidelines recommended for reporting animal research, and Sex and Gender in Research

Laboratory animals	Female and male C57BL/6J mice, sourced from Charles Rivers Laboratories, were used at 5 to 6 weeks of age.<br>Female BALB/c mice, sourced from Charles Rivers Laboratories, were used at 5 to 6 weeks of age.<br>Mice were maintained under specific-pathogen-free conditions and housed at 23°C and 40–50% humidity with a 12h light/12h dark schedule in accordance with Portuguese law, the European Commission recommendations on housing and care of animals and the Federation of European Laboratory Animal Science Associations guidelines category B.
Wild animals	No wild animals were used in this study
Reporting on sex	Both male and female mice were used in this study.
Field-collected samples	No field-collected samples were used in this study
Ethics oversight	Animal experimentation protocols were approved by the institutional animal welfare body – ORBEA-GIMM – and by the Direcção-Geral de Alimentação e Veterinária. All procedures were performed in strict accordance with the Directive 2010/63/EU for the care and use of animals for scientific purposes.

Note that full information on the approval of the study protocol must also be provided in the manuscript.

Plants

Seed stocks

Report on the source of all seed stocks or other plant material used. If applicable, state the seed stock centre and catalogue number. If plant specimens were collected from the field, describe the collection location, date and sampling procedures.

Novel plant genotypes

Describe the methods by which all novel plant genotypes were produced. This includes those generated by transgenic approaches, gene editing, chemical/radiation-based mutagenesis and hybridization. For transgenic lines, describe the transformation method, the number of independent lines analyzed and the generation upon which experiments were performed. For gene-edited lines, describe the editor used, the endogenous sequence targeted for editing, the targeting guide RNA sequence (if applicable) and how the editor was applied.

Authentication

Describe any authentication procedures for each seed stock used or novel genotype generated. Describe any experiments used to assess the effect of a mutation and, where applicable, how potential secondary effects (e.g. second site T-DNA insertions, mosiacism, off-target gene editing) were examined.

Flow Cytometry

Plots

Confirm that:

☒ The axis labels state the marker and fluorochrome used (e.g. CD4-FITC).

☒ The axis scales are clearly visible. Include numbers along axes only for bottom left plot of group (a 'group' is an analysis of identical markers).

☒ All plots are contour plots with outliers or pseudocolor plots.

☒ A numerical value for number of cells or percentage (with statistics) is provided.

Methodology

Sample preparation

Leukocytes: Mice were euthanized at indicated time points, transcardially perfused, and livers and spleens were collected and homogenized in PBS. Liver-infiltrating leukocytes were isolated in a 35% Percoll density gradient, after which erythrocytes were lysed. Ex-vivo cytokine production was assessed following culture with phorbol 12-myristate 13-acetate (PMA) and ionomycin for 4 hours at 37°C, 5% CO2, in the presence of brefeldin A. An incubation at 4ºC with a live/dead fixable dye diluted in PBS was performed to stain dead cells prior to surface or intracellular staining After, Fc receptors were blocked, and liver-infiltrating leukocytes and splenocytes were labeled with monoclonal antibodies diluted in staining buffer (PBS with 2% FBS) and staining performed at 4ºC. Intracellular staining to evaluate cytokine production was performed after cell fixation and permeabilization using the FoxP3/Transcription Factor Staining Kit, according to the manufacturer's instructions.

Parasitemia: Blood samples were collected from the tail vein in PBS and incubated for 10 minutes at 37ºC with monoclonal antibodies specific for Ter-119, CD45, and Hoechst 33342, all diluted in PBS.

Hepatocytes: Mice received 160ug of rhodamine-labeled Dextran (10,000 Da) diluted in PBS immediately before infection with sporozoites. Two hours after, livers were perfused in situ through vena cava with 15mL of perfusion buffer pre-warmed at 42ºC. Following, livers were perfused with 25mL of digestion buffer, supplemented with Liberase Blendzyme 3 recombinant collagenase, pre-warmed at 42ºC. After perfusion, liver tissue was gently dispersed in a single cell suspension, passed through a 100um filter, and liver parenchyma cells isolated in a 50% Percoll density gradient, centrifuged at 70g for 10 minutes. To evaluate in vivo cell wounding, hepatic single cell suspensions were stained with a monoclonal antibody specific for CD31 and Live/Dead Fixable Violet Dead Cell.

Circulating lymphocytes: To evaluate CD8 T cell depletion, blood samples were collected from the tail vein in PBS and erythrocyte lysis was performed. Leukocytes were incubated for 20 minutes at 4ºC with monoclonal antibodies specific for CD45, CD3, CD4 and CD8. Prior, Fc receptors were blocked using a monoclonal antibody specific for CD16/CD32. All antibodies were diluted in PBS.

Instrument

Samples were acquired in a 5-laser Cytek Aurora (Cytek Biosciences) or a BD LSRFortessa (BD Biosciences) analyzer.

Software

Samples were aquired using SpectroFlo software V3.3.0 (Cytek Biosciences) or FACSDiva software V6.2.0 (BD Biosciences). Samples were analysed using FlowJo V10.10.0 software (Tree Star Inc.). For the hierarchical unsupervised analysis, the Peak Extraction and Cleaning Oriented Quality Control (PeacoQC) plugin was used to exclude abnormal events. The UMAP plugin was then used to perform dimensional reduction, followed by unsupervised hierarchical clustering using the FlowSOM plugin.

Cell population abundance

No cell sortings were performed in this study

Gating strategy

Gating strategy used for downstream cell clustering of liver-isolated leukocytes: Leukocytes were gated using the forward scatter (FSC-A) and side scatter (SSC-A) parameters, cell aggregates were excluded using FSC-A vs. FSC-W, and live leukocytes were selected based on live/dead discrimination in a CD45 vs. Live dead dye plot. Lymphocytes and natural killer T cells were selected for concatenation based on the expression of CD3 and NK1.1 in a CD3 vs. NK1.1 plot. CD3- leukocytes were further gated based on the expression of CD11b and/or NK1.1 for concatenation and analysis of myeloid and natural killer subsets.

Gating strategy used for analysis of liver immune infiltrates and Plasmodium-specific CD8 tissue resident T cells: Leukocytes

were first gated based on forward scatter (FSC-A) and side scatter (SSC-A) parameters, and cell aggregates were excluded using FSC-A vs. FSC-W. Live leukocytes were selected by gating on CD45 cells while excluding dead cells using a live dead dye. T cells were then identified based on TCRβ and CD3 expression in a CD3 vs. TCRβ plot. CD3 TCRβ T cells were further subdivided into $CD4^{+}$ and $CD8^{+}$ subsets using CD4 vs. CD8 plot. For downstream phenotypic and functional analysis, activated CD8 T cells were discriminated in a CD44 vs. CD62L plot, and analyzed for the expression of KLRG1 and CD69. Tissue-residency phenotype was assessed by the expression of CXCR6, CXCR3, CD127 and PD-1, as well as by the lack of CX3CR1 expression. All memory CD8 T cell subsets were analysed for Plasmodium specificity using a PbTRAP vs. PbRPL6 gate. Functional profiling was performed by assessing the expression of IFN-γ and Granzyme B.

Gating strategy used to analyze the frequency of infected erythrocytes (parasitemia): Erythrocytes were gated using the forward scatter (FSC-A) and side scatter (SSC-A) parameters, cell aggregates were excluded using FSC-A vs. FSC-W, and erythrocytes were selected based on Ter119 expression in a CD45 vs. TER119 plot. DNA content using Hoechst 33342 discriminated between infected and non-infected erythrocytes in a Hoechst 33342 vs. CD45 plot.

Gating strategy used to analyze the frequency of traversed liver parenchyma cells: Liver parenchyma cells were gated using the forward scatter (FSC-A) and side scatter (SSC-A) parameters, cell aggregates were excluded using FSC-A vs. FSC-W, endothelial cells were excluded based on CD31 expression, and live parenchyma cells were selected in a CD31 vs. live/dead dye plot. Traversed cells were identified based on their content of rhodamine dextran in a GFP vs. rhodamine dextran plot.

Gating strategy used to evaluate the depletion of CD8 T cells: Circulating leukocytes, or isolated from livers or spleens, were gated using the forward scatter (FSC-A) and side scatter (SSC-A) parameters, cell aggregates were excluded using FSC-A vs. FSC-W, and lymphocytes were selected based on CD45 and CD3 expression. Lymphocytes were further gated according to the expression of CD8 and CD4.

☒ Tick this box to confirm that a figure exemplifying the gating strategy is provided in the Supplementary Information.
